# Supplementary material for: Idealized 3D Auxetic Mechanical Metamaterial: An Analytical, Numerical, and Experimental Study
Source: Materials (Basel). 2021 Feb 20;14(4):993. doi: 10.3390/ma14040993 (PMC7923447; doi:10.3390/ma14040993)
Supplement: Supplementary file 1 [file materials-14-00993-s001.pdf]

Supplementary materials

S1. More details on Materials and Methods

S.1.1. More Geometrical Schematics

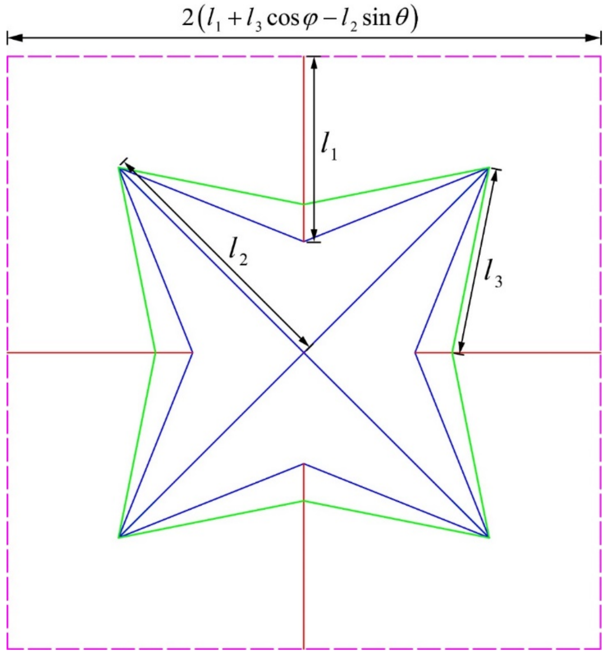

Figure S1. The side view of the re-entrant unit cell and its surrounded square.

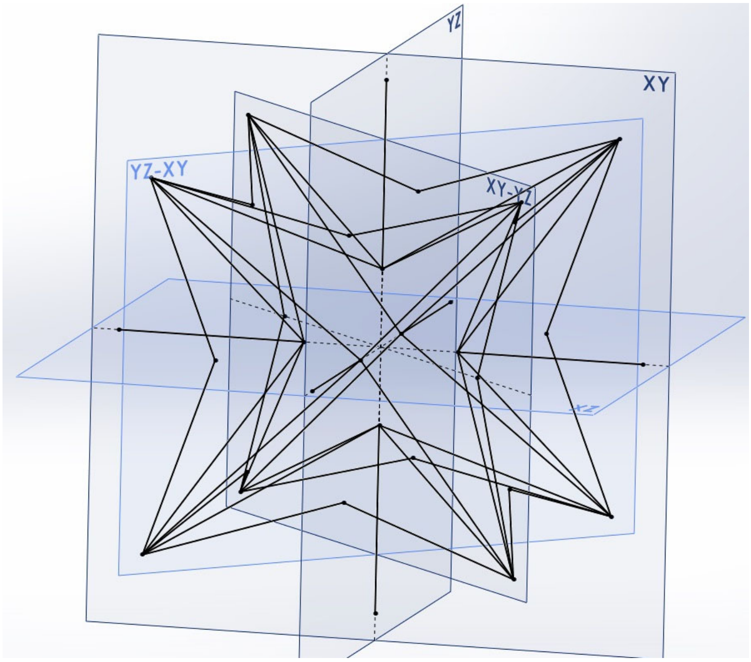

Figure S2. The symmetry planes of 3D re-entrant unit cell.

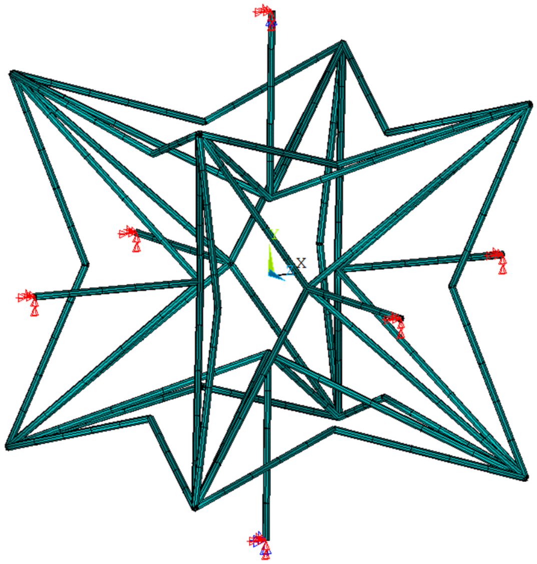

Figure S3. Applied repetitive boundary condition to a single reentrant unit cell.

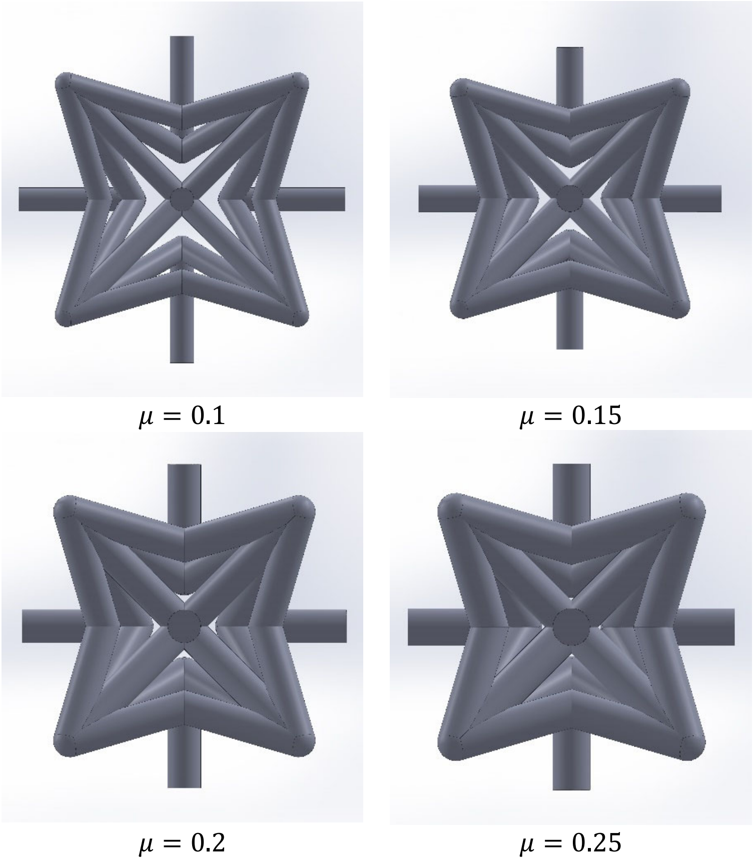

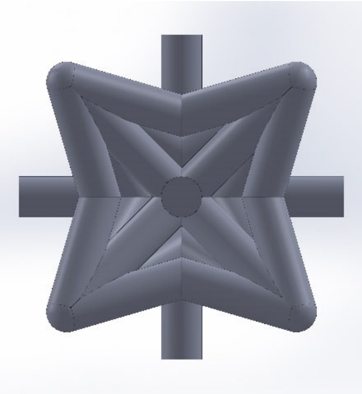

$\mu = 0.3$

Figure S4. SolidWorks CAD models of additive manufactured specimens.

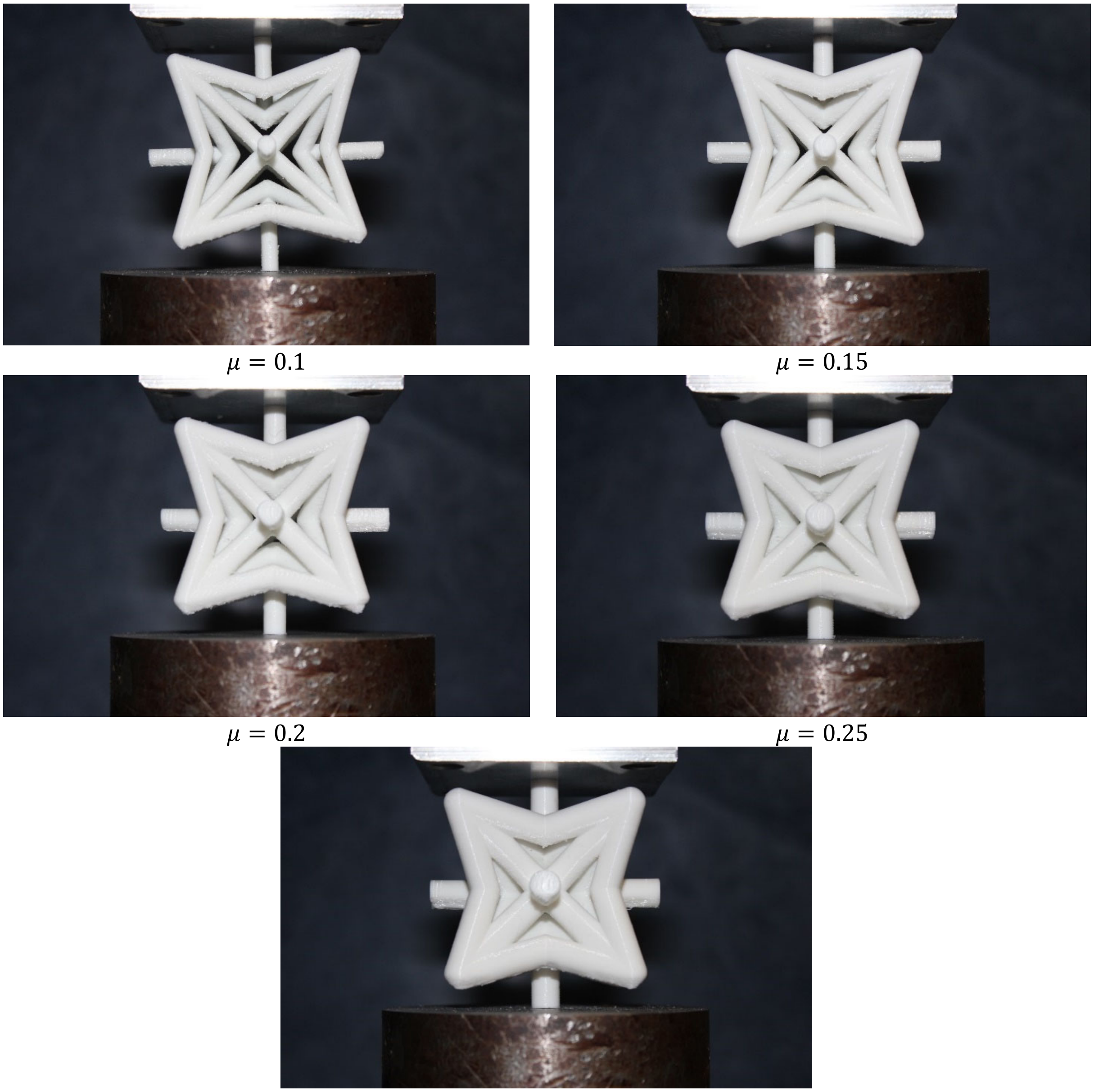

$\mu = 0.1$ $\mu = 0.15$

$\mu = 0.2$ $\mu = 0.25$

$\mu = 0.3$

Figure S5. 3D-printed specimens in a compression test.

S.1.2. Calculation of Other Elements of the Stiffness Matrix

a) The Third DOF: $q_3 = 1$

During this deformation, point C of struts CD displaces vertically and parallel to the Y direction. This degree of freedom (DOF) deforms struts CD only. Struts CD are deformed in longitudinal and transverse directions and the resultant forces and moments in vertices C and D of struts CD determine the $K_{33}, K_{34}, K_{35}, K_{36}, K_{310}$ and K_{311} elements of the stiffness matrix. Similar to what was described in the manuscript for $\boldsymbol{q}_2$, by projecting the resultant elongation force vector $\left((\boldsymbol{q}_3.\boldsymbol{u}_{C_1D_1})\boldsymbol{u}_{C_1D_1}\right)S_3$ and the transverse deformation force vector $(\boldsymbol{q}_3 - (\boldsymbol{q}_3.\boldsymbol{u}_{C_1D_1})\boldsymbol{u}_{C_1D_1})T_3$ of strut CD in the $\boldsymbol{q}_3$ direction (i.e., Y direction), and by solving the equilibrium of forces in this direction at point C, the element K_{33} of the stiffness matrix could be obtained as follows:

$$\sum F_{y,C_1} = 0 \rightarrow \frac{Q_3}{8} - 2\boldsymbol{q}_3.\left((\boldsymbol{q}_3.\boldsymbol{u}_{C_1D_1})\boldsymbol{u}_{C_1D_1}\right)S_3 - 2\boldsymbol{q}_3.(\boldsymbol{q}_3 - (\boldsymbol{q}_3.\boldsymbol{u}_{C_1D_1})\boldsymbol{u}_{C_1D_1})T_3 = 0$$

$$\rightarrow K_{33} = Q_3 = 8\sin^2\varphi\, S_3 + 8(\cos^2\varphi + 1)$$

Similarly, by projecting the resultant elongation force vector $\left((\boldsymbol{q}_3.\boldsymbol{u}_{C_1D_1})\boldsymbol{u}_{C_1D_1}\right)S_3$ and the transverse deformation force vector $(\boldsymbol{q}_3 - (\boldsymbol{q}_3.\boldsymbol{u}_{C_1D_1})\boldsymbol{u}_{C_1D_1})T_3$ of strut CD in the q_4 direction (i.e., Z direction) and solving the equilibrium of forces in this direction at point C, the element K_{34} of the stiffness matrix could be obtained as follows:

$$\sum F_{z,C_1} = 0 \rightarrow \frac{Q_4}{8} - 2\boldsymbol{q}_4.\left((\boldsymbol{q}_3.\boldsymbol{u}_{C_1D_1})\boldsymbol{u}_{C_1D_1}\right)S_3 - 2\boldsymbol{q}_4.(\boldsymbol{q}_3 - (\boldsymbol{q}_3.\boldsymbol{u}_{C_1D_1})\boldsymbol{u}_{C_1D_1})T_3 = 0$$

$$\rightarrow K_{34} = Q_4 = 8\sin^2\varphi\,(S_3 - T_3)$$

By considering the reactions of force vectors (elongation and transverse deformation) at point D and solving the equilibrium of forces in the $\boldsymbol{q}_5$ and $\boldsymbol{q}_6$ directions at point D, the elements K_{35} and K_{36} of the stiffness matrix could be derived as follows:

$$\sum F_{y,D_1} = 0 \rightarrow \frac{Q_5}{8} + 2\boldsymbol{q}_5.\left((\boldsymbol{q}_3.\boldsymbol{u}_{C_1D_1})\boldsymbol{u}_{C_1D_1}\right)S_3 + 2\boldsymbol{q}_5.(\boldsymbol{q}_3 - (\boldsymbol{q}_3.\boldsymbol{u}_{C_1D_1})\boldsymbol{u}_{C_1D_1})T_3 = 0$$

$$\rightarrow K_{35} = Q_5 = 8\sin^2\varphi\, S_3 + 8(\cos^2\varphi + 1)$$

and:

$$\sum F_{q_6,D_1} = 0 \rightarrow \frac{Q_6}{8} + 2\boldsymbol{q}_6.\left((\boldsymbol{q}_3.\boldsymbol{u}_{C_1D_1})\boldsymbol{u}_{C_1D_1}\right)S_3 + 2\boldsymbol{q}_6.(\boldsymbol{q}_3 - (\boldsymbol{q}_3.\boldsymbol{u}_{C_1D_1})\boldsymbol{u}_{C_1D_1})T_3 = 0$$

$$\rightarrow K_{36} = Q_6 = 4\left(\sin 2\varphi + \sqrt{2}\sin^2\varphi\right)(S_3 - T_3)$$

Because $\boldsymbol{q}_{10}$ and $\boldsymbol{q}_{11}$ are rotational DOFs, the elements K_{310} and K_{311} can be obtained by solving the equilibrium of moments at points D and C, respectively. By projecting the moment vector, created by transverse deformation of point C of strut CD, in the $\boldsymbol{q}_{11}$ direction and solving the equilibrium of moments at point C, the element K_{310} could be obtained. By doing the same at point D in the $\boldsymbol{q}_{10}$ direction, the element K_{311} could be obtained as follows:

$$\sum M_{q_{10},D_1} = 0 \rightarrow \frac{Q_{10}}{8} - 2\boldsymbol{q}_{10}.\left(\boldsymbol{u}_{C_1D_1} \times (\boldsymbol{q}_3 - (\boldsymbol{q}_3.\boldsymbol{u}_{C_1D_1})\boldsymbol{u}_{C_1D_1})\right)V_3 = 0 \rightarrow K_{310} = Q_{10}$$

$$= -8\left(\sin\varphi + \sqrt{2}\cos\varphi\right)V_3$$

$$\sum M_{q_{11},C_1} = 0 \rightarrow \frac{Q_{11}}{8} - 2\boldsymbol{q}_{11}.\left(\boldsymbol{u}_{C_1D_1} \times (\boldsymbol{q}_3 - (\boldsymbol{q}_3.\boldsymbol{u}_{C_1D_1})\boldsymbol{u}_{C_1D_1})\right)V_3 = 0 \rightarrow K_{311} = Q_{11}$$

$$= -8\left(\sqrt{2}\sin\varphi\right)V_3$$

b) The Forth DOF: $q_4 = 1$

This degree of freedom (DOF) is similar to q_3 and only deforms strut CD in its longitudinal and transverse directions. The resultant forces and moments at vertices C and D of strut CD determine the elements K_{43}, K_{44}, K_{45}, K_{46}, K_{410} and K_{411} of the stiffness matrix. The procedure of extracting the noted elements of the stiffness matrix is similar to the procedure described in the manuscript for the third DOF. The equations of equilibrium are as follows:

$$\sum F_{y,C_1} = 0 \rightarrow -\frac{Q_4}{8} + 2\boldsymbol{q}_4.\left((\boldsymbol{q}_4.\boldsymbol{u}_{C_1D_1})\boldsymbol{u}_{C_1D_1}\right)S_3 + 2\boldsymbol{q}_4.\left(\boldsymbol{q}_4 - (\boldsymbol{q}_4.\boldsymbol{u}_{C_1D_1})\boldsymbol{u}_{C_1D_1}\right)T_3 = 0 \rightarrow K_{44} = Q_4 = 8\sin^2\varphi\, S_3 + 8(\cos^2\varphi + 1)$$

$$\sum F_{y,D_1} = 0 \rightarrow -\frac{Q_5}{8} - 2\boldsymbol{q}_5.\left((\boldsymbol{q}_4.\boldsymbol{u}_{C_1D_1})\boldsymbol{u}_{C_1D_1}\right)S_3 - 2\boldsymbol{q}_5.\left(\boldsymbol{q}_4 - (\boldsymbol{q}_4.\boldsymbol{u}_{C_1D_1})\boldsymbol{u}_{C_1D_1}\right)T_3 = 0 \rightarrow K_{45} = Q_5 = 8\sin^2\varphi\,(S_3 - T_3)$$

$$\sum F_{q_6,D_1} = 0 \rightarrow -\frac{Q_6}{8} - 2\boldsymbol{q}_6.\left((\boldsymbol{q}_4.\boldsymbol{u}_{C_1D_1})\boldsymbol{u}_{C_1D_1}\right)S_3 - 2\boldsymbol{q}_6.\left(\boldsymbol{q}_4 - (\boldsymbol{q}_4.\boldsymbol{u}_{C_1D_1})\boldsymbol{u}_{C_1D_1}\right)T_3 = 0 \rightarrow K_{46} = Q_6 = 4\left(\sin 2\varphi + \sqrt{2}\sin^2\varphi\right)S_3 + 2\left(3\sqrt{2} - 2\sin 2\varphi + \sqrt{2}\,cos\,2\varphi\right)S_3$$

$$\sum M_{q_{10},D_1} = 0 \rightarrow -\frac{Q_{10}}{8} + 2\boldsymbol{q}_{10}.\left(\boldsymbol{u}_{C_1D_1} \times \left(\boldsymbol{q}_4 - (\boldsymbol{q}_4.\boldsymbol{u}_{C_1D_1})\boldsymbol{u}_{C_1D_1}\right)\right)V_3 = 0 \rightarrow K_{410} = Q_{10} = 8\sin\varphi\, V_3$$

$$\sum M_{q_{11},C_1} = 0 \rightarrow -\frac{Q_{11}}{8} + 2\boldsymbol{q}_{11}.\left(\boldsymbol{u}_{C_1D_1} \times \left(\boldsymbol{q}_4 - (\boldsymbol{q}_4.\boldsymbol{u}_{C_1D_1})\boldsymbol{u}_{C_1D_1}\right)\right)V_3 = 0 \rightarrow K_{411} = Q_{11} = 8\left(\sqrt{2}\sin\varphi\right)V_3$$

c) The Sixth DOF: $q_5 = 1$

This DOF creates one of the most complex deformations in the structure. Due to $\boldsymbol{q}_5$, vertex D displaces vertically in the Y direction and affects four strut types (DC, DE, DG, and DB) showed in Figure S5. This leads to only two zero elements of the fifth column of the stiffness matrix K_{51} and K_{58}, and the other elements of the fifth are non-zero. When vertex D is displaced by unity, axial contraction/expansion and lateral deformations are applied to all the struts connected to point D.

The value of axial contraction/elongation of struts DC, DE, DG, and DB can be found by projecting the vector $\boldsymbol{q}_5$ on the unit vectors of each strut ($\boldsymbol{u}_{C_1D_1}, \boldsymbol{u}_{E_1D_1}, \boldsymbol{u}_{B_1D_1}$ and $\boldsymbol{u}_{G_1D_1}$). Then, by multiplying the resultant axial forces of each strut (S_2 and S_3), the axial force vectors could be obtained. The value of lateral deformation of struts DC, DE, DG, and DB can be found by subtracting the axial contraction/elongation vector from the vector $\boldsymbol{q}_5$, i.e., ($\boldsymbol{q}_5 - (\boldsymbol{q}_5.\boldsymbol{u}_{C_1D_1})\boldsymbol{u}_{C_1D_1}, \boldsymbol{q}_5 - (\boldsymbol{q}_5.\boldsymbol{u}_{E_1D_1})\boldsymbol{u}_{E_1D_1}$ and $\boldsymbol{q}_5 - (\boldsymbol{q}_5.\boldsymbol{u}_{B_1D_1})\boldsymbol{u}_{B_1D_1}, \boldsymbol{q}_5 - (\boldsymbol{q}_5.\boldsymbol{u}_{G_1D_1})\boldsymbol{u}_{G_1D_1}$. Afterward, by multiplying the resultant lateral forces of each strut (T_2 and T_3), the lateral force vectors could be calculated. It is worth noting that the terms related to struts CD and ED must be multiplied by two because two of such struts are connected to vertex D. Finally, by projecting all the resultant axial and lateral forces in the $\boldsymbol{q}_5$ direction and writing the force equilibrium equations in the $\boldsymbol{q}_5$ direction, the element K_{55} of the stiffness matrix can be obtained as follows:

$$\sum F_{y,D_1} = 0 \rightarrow \frac{Q_5}{8} - 2\boldsymbol{q}_5.\left((\boldsymbol{q}_5.\boldsymbol{u}_{C_1D_1})\boldsymbol{u}_{C_1D_1}\right)S_3 - 2\boldsymbol{q}_5.\left(\boldsymbol{q}_5 - (\boldsymbol{q}_5.\boldsymbol{u}_{C_1D_1})\boldsymbol{u}_{C_1D_1}\right)T_3$$
$$- 2\boldsymbol{q}_5.\left((\boldsymbol{q}_5.\boldsymbol{u}_{E_1D_1})\boldsymbol{u}_{E_1D_1}\right)S_2 - 2\boldsymbol{q}_5.\left(\boldsymbol{q}_5 - (\boldsymbol{q}_5.\boldsymbol{u}_{E_1D_1})\boldsymbol{u}_{E_1D_1}\right)T_2$$
$$- \boldsymbol{q}_5.\left((\boldsymbol{q}_5.\boldsymbol{u}_{B_1D_1})\boldsymbol{u}_{B_1D_1}\right)S_2 - \boldsymbol{q}_5.\left(\boldsymbol{q}_5 - (\boldsymbol{q}_5.\boldsymbol{u}_{B_1D_1})\boldsymbol{u}_{B_1D_1}\right)T_2$$
$$- \boldsymbol{q}_5.\left((\boldsymbol{q}_5.\boldsymbol{u}_{G_1D_1})\boldsymbol{u}_{G_1D_1}\right)S_3 - \boldsymbol{q}_5.\left(\boldsymbol{q}_5 - (\boldsymbol{q}_5.\boldsymbol{u}_{G_1D_1})\boldsymbol{u}_{G_1D_1}\right)T_3 = 0$$

$$\rightarrow K_{55} = Q_5 = 4(2\sin^2\theta\, S_2 + 2S_3 + 2\sin^2\theta\,(S_2 - T_2) + 5T_2 + cos\,2\theta\, T_2 + 4T_3)$$

Obtaining the element K_{56} of the stiffness matrix is similar to what was described for K_{55}, but now in the $\boldsymbol{q}_6$ direction, which is calculated as

$$\sum F_{q_6,D_1} = 0 \rightarrow \frac{Q_6}{8} - 2\boldsymbol{q}_6.\left((\boldsymbol{q}_5.\boldsymbol{u}_{C_1D_1})\boldsymbol{u}_{C_1D_1}\right)S_3 - 2\boldsymbol{q}_6.\left(\boldsymbol{q}_5 - (\boldsymbol{q}_5.\boldsymbol{u}_{C_1D_1})\boldsymbol{u}_{C_1D_1}\right)T_3$$
$$- 2\boldsymbol{q}_5.\left((\boldsymbol{q}_6.\boldsymbol{u}_{E_1D_1})\boldsymbol{u}_{E_1D_1}\right)S_2 - 2\boldsymbol{q}_6.\left(\boldsymbol{q}_5 - (\boldsymbol{q}_5.\boldsymbol{u}_{E_1D_1})\boldsymbol{u}_{E_1D_1}\right)T_2$$
$$- \boldsymbol{q}_6.\left((\boldsymbol{q}_5.\boldsymbol{u}_{B_1D_1})\boldsymbol{u}_{B_1D_1}\right)S_2 - \boldsymbol{q}_6.\left(\boldsymbol{q}_5 - (\boldsymbol{q}_5.\boldsymbol{u}_{B_1D_1})\boldsymbol{u}_{B_1D_1}\right)T_2$$
$$- \boldsymbol{q}_6.\left((\boldsymbol{q}_5.\boldsymbol{u}_{G_1D_1})\boldsymbol{u}_{G_1D_1}\right)S_3 - \boldsymbol{q}_6.\left(\boldsymbol{q}_5 - (\boldsymbol{q}_5.\boldsymbol{u}_{G_1D_1})\boldsymbol{u}_{G_1D_1}\right)T_3 = 0$$

$$\rightarrow K_{56} = Q_6 = 8\,sin\,2\theta\,(S_2 - T_2) + 4\sqrt{2}\cos^2\theta\,(S_2 - T_2) + 4(2\,sin\,2\varphi + \sqrt{2}\sin^2\varphi)(S_3 - T_3)$$

The resultant forces due to $\boldsymbol{q}_5$ create reaction forces at point B of strut DB. Therefore, solving the equilibrium of forces at point B in the $\boldsymbol{q}_7$ direction gives the element K_{57} of the fifth column of the stiffness matrix. The reaction forces must be multiplied by four due to the connection of four DB struts to vertex B, which is

$$\sum F_{y,B_1} = 0 \rightarrow \frac{Q_7}{2} + 4\boldsymbol{q}_7.\left((\boldsymbol{q}_5.\boldsymbol{u}_{B_1D_1})\boldsymbol{u}_{B_1D_1}\right)S_2 + 4\boldsymbol{q}_7.\left(\boldsymbol{q}_5 - (\boldsymbol{q}_5.\boldsymbol{u}_{B_1D_1})\boldsymbol{u}_{B_1D_1}\right)T_2 = 0$$

$$\rightarrow K_{57} = Q_7 = 8(\sin^2\theta\,S_2 + \cos^2\theta\,T_2)$$

The resultant forces created by $\boldsymbol{q}_5$ form reaction forces at point G of strut DG. Therefore, by solving the equations of equilibrium of forces at point G in the $\boldsymbol{q}_9$ direction, the element K_{59} of the stiffness matrix can be obtained. The reaction must be multiplied by two because there are two struts DB connected to point G, which is calculated as

$$\sum F_{q_9,E_1} = 0 \rightarrow \frac{Q_9}{4} + 2\boldsymbol{q}_9.\left((\boldsymbol{q}_5.\boldsymbol{u}_{G_1D_1})\boldsymbol{u}_{G_1D_1}\right)S_3 - 2\boldsymbol{q}_9.\left(\boldsymbol{q}_5 - (\boldsymbol{q}_5.\boldsymbol{u}_{G_1D_1})\boldsymbol{u}_{G_1D_1}\right)T_3 = 0$$

$$\rightarrow K_{59} = Q_9 = 4\,sin\,2\varphi\,(T_3 - S_3)$$

The elements K_{510} and K_{511} could be derived by solving the equations of moment equilibrium at vertices D and C, respectively. As a result of $\boldsymbol{q}_5$, four moment vectors are created by deformations of struts DC, DE, DG, and DB. By projecting these moment vectors on the $\boldsymbol{q}_{10}$ direction and solving the equilibrium of moments in the same direction, element K_{510} of the stiffness matrix can be obtained as

$$\sum M_{q_{10},D_1} = 0$$
$$\rightarrow \frac{Q_{10}}{8} + 2\boldsymbol{q}_{10}.\left(\boldsymbol{u}_{C_1D_1} \times \left(\boldsymbol{q}_5 - (\boldsymbol{q}_5.\boldsymbol{u}_{C_1D_1})\boldsymbol{u}_{C_1D_1}\right)\right)V_3$$
$$+ 2\boldsymbol{q}_{10}.\left(\boldsymbol{u}_{E_1D_1} \times \left(\boldsymbol{q}_5 - (\boldsymbol{q}_5.\boldsymbol{u}_{E_1D_1})\boldsymbol{u}_{E_1D_1}\right)\right)V_2$$
$$+ \boldsymbol{q}_{10}.\left(\boldsymbol{u}_{B_1D_1} \times \left(\boldsymbol{q}_5 - (\boldsymbol{q}_5.\boldsymbol{u}_{B_1D_1})\boldsymbol{u}_{B_1D_1}\right)\right)V_2$$
$$+ \boldsymbol{q}_{10}.\left(\boldsymbol{u}_{G_1D_1} \times \left(\boldsymbol{q}_5 - (\boldsymbol{q}_5.\boldsymbol{u}_{G_1D_1})\boldsymbol{u}_{G_1D_1}\right)\right)V_3 = 0$$

$$\rightarrow K_{510} = Q_{10} = -8\sqrt{2}(sin\,\theta + \sqrt{2}\,cos\,\theta)V_2 - 8(\sqrt{2}\,cos\,\varphi + 2\,sin\,\varphi)V_3$$

The deformations of strut DC due to $\boldsymbol{q}_5$ DOF creates reaction moments at points C and the equilibrium of moments at this point and $\boldsymbol{q}_{11}$ direction yields K_{511} as follows:

$$\sum M_{q_{11},C_1} = 0 \rightarrow \frac{Q_{11}}{8} + 2\boldsymbol{q}_{11}.\left(\boldsymbol{u}_{C_1D_1} \times \left(\boldsymbol{q}_5 - (\boldsymbol{q}_5.\boldsymbol{u}_{C_1D_1})\boldsymbol{u}_{C_1D_1}\right)\right)V_3 = 0$$
$$\rightarrow K_{511} = Q_{11} = -8\sqrt{2}\,sin\,\varphi\,V_3$$

d) Sixth DOF: $q_6 = 1$

This DOF is similar to q_5 and deforms struts DC, DE, DG, and DB in their axial and lateral directions. The resultant forces and moments at vertices C and D of strut CD determine the $K_{62}, K_{63}, K_{64}, K_{65}, K_{66}, K_{67}, K_{610}$ and K_{611} elements of the stiffness matrix. The procedure of extracting the elements of the stiffness matrix is similar to the procedure of the fifth DOF, and the equations of equilibrium are as follows:

$$\sum F_{q_6,D_1} = 0 \rightarrow -\frac{Q_6}{8} + 2\boldsymbol{q}_6.\left((\boldsymbol{q}_6.\boldsymbol{u}_{C_1D_1})\boldsymbol{u}_{C_1D_1}\right)S_3 + 2\boldsymbol{q}_6.\left(\boldsymbol{q}_6 - (\boldsymbol{q}_6.\boldsymbol{u}_{C_1D_1})\boldsymbol{u}_{C_1D_1}\right)T_3 + 2\boldsymbol{q}_6.\left((\boldsymbol{q}_6.\boldsymbol{u}_{E_1D_1})\boldsymbol{u}_{E_1D_1}\right)S_2 + 2\boldsymbol{q}_6.\left(\boldsymbol{q}_6 - (\boldsymbol{q}_6.\boldsymbol{u}_{E_1D_1})\boldsymbol{u}_{E_1D_1}\right)T_2 + \boldsymbol{q}_6.\left((\boldsymbol{q}_6.\boldsymbol{u}_{B_1D_1})\boldsymbol{u}_{B_1D_1}\right)S_2 + \boldsymbol{q}_6.\left(\boldsymbol{q}_6 - (\boldsymbol{q}_6.\boldsymbol{u}_{B_1D_1})\boldsymbol{u}_{B_1D_1}\right)T_2 + \boldsymbol{q}_6.\left((\boldsymbol{q}_6.\boldsymbol{u}_{G_1D_1})\boldsymbol{u}_{G_1D_1}\right)S_3 + \boldsymbol{q}_6.\left(\boldsymbol{q}_6 - (\boldsymbol{q}_6.\boldsymbol{u}_{G_1D_1})\boldsymbol{u}_{G_1D_1}\right)T_3 = 0$$

$$\rightarrow K_{66} = Q_6 = 2(4\sin^2\theta\, S_2 + (5 - cos\,2\varphi + 2\sqrt{2}\,sin\,2\varphi)S_3 + 2\sqrt{2}\,sin\,2\theta\,(S_2 - T_2) + 6\sin^2\theta\,(S_2 - T_2) + 10T_2 + 2\,cos\,2\theta\,T_2 + (7 + cos\,2\varphi - 2\sqrt{2}\,sin\,2\varphi)T_3)$$

$$\sum F_{z,B_1} = 0 \rightarrow -\frac{Q_7}{2} - 4\boldsymbol{q}_7.\left((\boldsymbol{q}_6.\boldsymbol{u}_{B_1D_1})\boldsymbol{u}_{B_1D_1}\right)S_2 - 4\boldsymbol{q}_7.\left(\boldsymbol{q}_6 - (\boldsymbol{q}_6.\boldsymbol{u}_{B_1D_1})\boldsymbol{u}_{B_1D_1}\right)T_2 = 0 \rightarrow K_{67} = Q_7 = 8\,sin\,2\theta\,(S_2 - T_2)$$

$$\sum F_{q_9,G_1} = 0 \rightarrow -\frac{Q_9}{4} - 2\boldsymbol{q}_9.\left((\boldsymbol{q}_6.\boldsymbol{u}_{G_1D_1})\boldsymbol{u}_{G_1D_1}\right)S_3 - 2\boldsymbol{q}_9.\left(\boldsymbol{q}_6 - (\boldsymbol{q}_6.\boldsymbol{u}_{G_1D_1})\boldsymbol{u}_{G_1D_1}\right)T_3 = 0 \rightarrow K_{69} = Q_9 = -8(\sin^2\varphi\,S_3 + \cos^2\varphi\,T_3)$$

$$\sum M_{q_{10},D_1} = 0 \rightarrow -\frac{Q_{10}}{8} - 2\boldsymbol{q}_{10}.\left(\boldsymbol{u}_{C_1D_1} \times \left(\boldsymbol{q}_6 - (\boldsymbol{q}_6.\boldsymbol{u}_{C_1D_1})\boldsymbol{u}_{C_1D_1}\right)\right)V_3 - 2\boldsymbol{q}_{10}.\left(\boldsymbol{u}_{E_1D_1} \times \left(\boldsymbol{q}_6 - (\boldsymbol{q}_6.\boldsymbol{u}_{E_1D_1})\boldsymbol{u}_{E_1D_1}\right)\right)V_2 - \boldsymbol{q}_{10}.\left(\boldsymbol{u}_{B_1D_1} \times \left(\boldsymbol{q}_6 - (\boldsymbol{q}_6.\boldsymbol{u}_{B_1D_1})\boldsymbol{u}_{B_1D_1}\right)\right)V_2 - \boldsymbol{q}_{10}.\left(\boldsymbol{u}_{G_1D_1} \times \left(\boldsymbol{q}_6 - (\boldsymbol{q}_6.\boldsymbol{u}_{G_1D_1})\boldsymbol{u}_{G_1D_1}\right)\right)V_3 = 0 \rightarrow K_{610} = Q_{10} = 8((sin\,\theta + \sqrt{2}\,cos\,\theta)V_2 + (cos\,\varphi + \sqrt{2}\,sin\,\varphi)V_3)$$

$$\sum M_{q_{11},C_1} = 0 \rightarrow -\frac{Q_{11}}{8} - 2\boldsymbol{q}_{11}.\left(\boldsymbol{u}_{C_1D_1} \times \left(\boldsymbol{q}_6 - (\boldsymbol{q}_6.\boldsymbol{u}_{C_1D_1})\boldsymbol{u}_{C_1D_1}\right)\right)V_3 = 0 \rightarrow K_{611} = Q_{11} = 8\,sin\,\varphi\,V_3$$

e) The Seventh DOF: $q_7 = 1$

This DOF is similar to q_2 and deforms struts AB and DB in their axial and lateral directions. The resultant forces and moments at vertices A, B, and D determine the elements $K_{78}, K_{77}, K_{75}, K_{76}$ and K_{710} of the stiffness matrix. The detailed equations are as follows:

$$\sum F_{y,B_1} = 0 \rightarrow -\frac{Q_7}{2} + \boldsymbol{q}_7.\left((\boldsymbol{q}_7.\boldsymbol{u}_{A_1B_1})\boldsymbol{u}_{A_1B_1}\right)S_1 + 4\boldsymbol{q}_7.\left((\boldsymbol{q}_7.\boldsymbol{u}_{B_1D_1})\boldsymbol{u}_{B_1D_1}\right)S_2 + 4\boldsymbol{q}_7.\left(\boldsymbol{q}_7 - (\boldsymbol{q}_7.\boldsymbol{u}_{B_1D_1})\boldsymbol{u}_{B_1D_1}\right)T_2 = 0 \rightarrow K_{77} = Q_7 = 2(S_1 + 4\sin^2\theta\,S_2 + 4\cos^2\theta\,T_2)$$

$$\sum F_{y,A_1} = 0 \rightarrow -\frac{Q_8}{2} - \boldsymbol{q}_8.\left((\boldsymbol{q}_7.\boldsymbol{u}_{A_1B_1})\boldsymbol{u}_{A_1B_1}\right)S_1 = 0 \rightarrow K_{78} = Q_8 = -2S_1$$

$$\sum M_{q_{10},D_1} = 0 \rightarrow -\frac{Q_{10}}{8} + \boldsymbol{q}_{10}.\left(\boldsymbol{u}_{B_1D_1} \times \left(\boldsymbol{q}_7 - (\boldsymbol{q}_7.\boldsymbol{u}_{B_1D_1})\boldsymbol{u}_{B_1D_1}\right)\right)V_2 = 0 \rightarrow K_{710} = Q_{10} = -8\sin\theta\,V_2$$

e) The Eighth DOF: $q_8 = 1$

This DOF is similar to $\boldsymbol{q}_1$ and deforms strut AB in its axial direction. The resultant forces at vertices A and B determine the elements K_{88} and K_{87} of the stiffness matrix. Therefore

$$\sum F_{y,A_1} = 0 \;\rightarrow\; -\frac{Q_8}{2} + \boldsymbol{q}_8.\left(\left(\boldsymbol{q}_8.\boldsymbol{u}_{A_1B_1}\right)\boldsymbol{u}_{A_1B_1}\right)S_1 = 0 \;\rightarrow K_{88} = Q_8 = 2S_1$$

f) The Ninth DOF: $q_9 = 1$

The derivations of this DOF is very similar to what is explained for $\boldsymbol{q}_3$ or $\boldsymbol{q}_4$. This DOF only deforms strut DG in its axial and lateral directions. The resultant forces and moments at vertices G and D determine the elements K_{88} and K_{87} of the stiffness matrix. The equilibrium equations are as follows:

$$\sum F_{q_9,G_1} = 0 \;\rightarrow\; -\frac{Q_9}{4} + 2\boldsymbol{q}_9.\left(\left(\boldsymbol{q}_9.\boldsymbol{u}_{G_1D_1}\right)\boldsymbol{u}_{G_1D_1}\right)S_3 + 2\boldsymbol{q}_9.\left(\boldsymbol{q}_9 - \left(\boldsymbol{q}_9.\boldsymbol{u}_{G_1D_1}\right)\boldsymbol{u}_{G_1D_1}\right)T_2 = 0 \;\rightarrow K_{99} = Q_9$$
$$= 8(\sin^2\varphi\, S_3 + \cos^2\varphi\, T_3)$$

$$\sum M_{q_{10},D_1} = 0 \;\rightarrow\; -\frac{Q_{10}}{8} + \boldsymbol{q}_{10}.\left(\boldsymbol{u}_{G_1D_1} \times \left(\boldsymbol{q}_9 - \left(\boldsymbol{q}_9.\boldsymbol{u}_{G_1D_1}\right)\boldsymbol{u}_{G_1D_1}\right)\right)V_2 = 0 \;\rightarrow K_{910} = Q_{10} = -8\sin\varphi\, V_3$$

g) The Eleventh DOF $q_{11} = 1$

The total procedure of calculating the stiffness matrix elements for this DOF is similar to that of $\boldsymbol{q}_{10}$, and even simpler than that, because this DOF only applies a rotation and torsion on point C of strut DC. Therefore

$$\sum M_{q_{11},C_1} = 0 \;\rightarrow\; -\frac{Q_{11}}{8} + 2\boldsymbol{q}_{11}.\left(\left(\boldsymbol{q}_{11}.\boldsymbol{u}_{C_1D_1}\right)\boldsymbol{u}_{C_1D_1}\right)J_3 + 2\boldsymbol{q}_{11}.\left(\boldsymbol{q}_{11} - \left(\boldsymbol{q}_{11}.\boldsymbol{u}_{C_1D_1}\right)\boldsymbol{u}_{C_1D_1}\right)U_3 = 0$$
$$\rightarrow K_{1111} = Q_{11} = 16(\cos^2\varphi J_3 + \sin^2\varphi U_3)$$

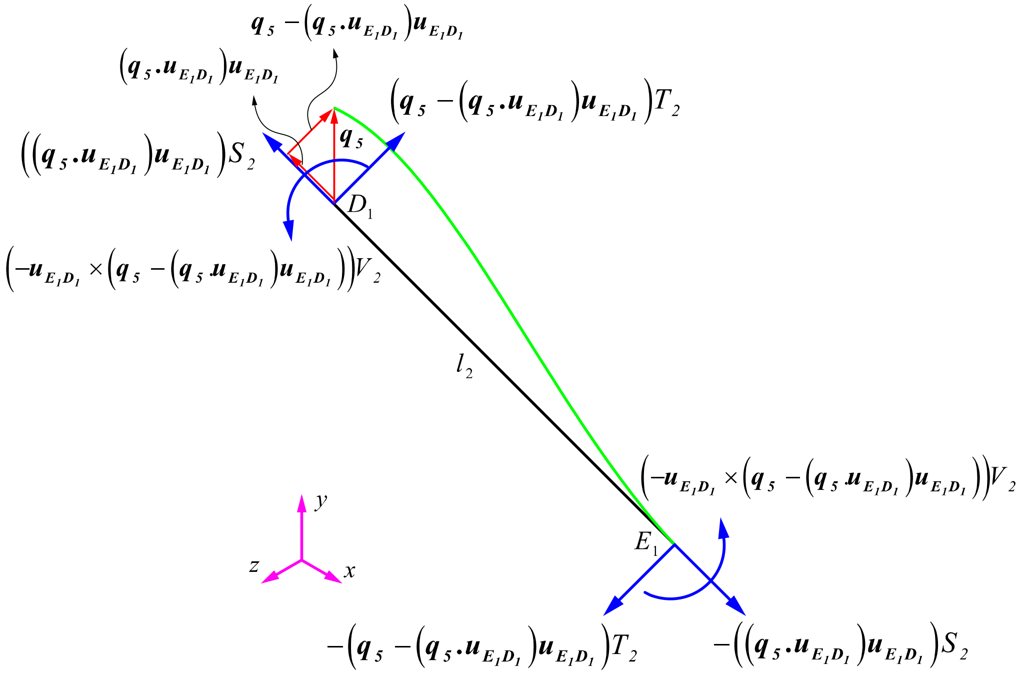

(a)

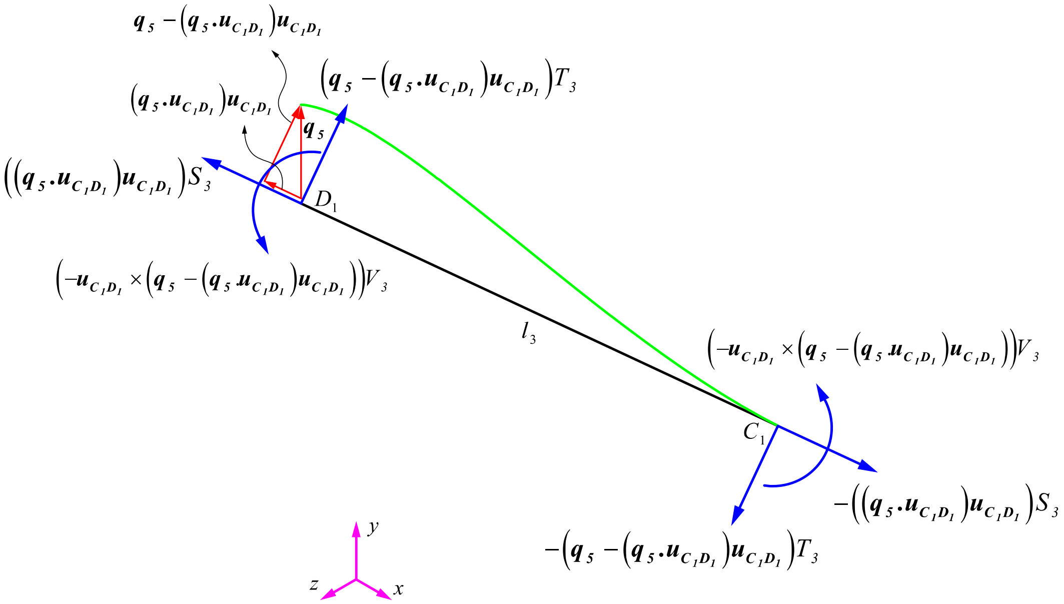

$q_5-(q_5.u_{C_1D_1})u_{C_1D_1}$
$(q_5-(q_5.u_{C_1D_1})u_{C_1D_1})T_3$
$(q_5.u_{C_1D_1})u_{C_1D_1}$
q_5
$((q_5.u_{C_1D_1})u_{C_1D_1})S_3$
D_1
$(-u_{C_1D_1}\times(q_5-(q_5.u_{C_1D_1})u_{C_1D_1}))V_3$
l_3
$(-u_{C_1D_1}\times(q_5-(q_5.u_{C_1D_1})u_{C_1D_1}))V_3$
C_1
$-((q_5.u_{C_1D_1})u_{C_1D_1})S_3$
$-(q_5-(q_5.u_{C_1D_1})u_{C_1D_1})T_3$
y
z
x

(**b**)

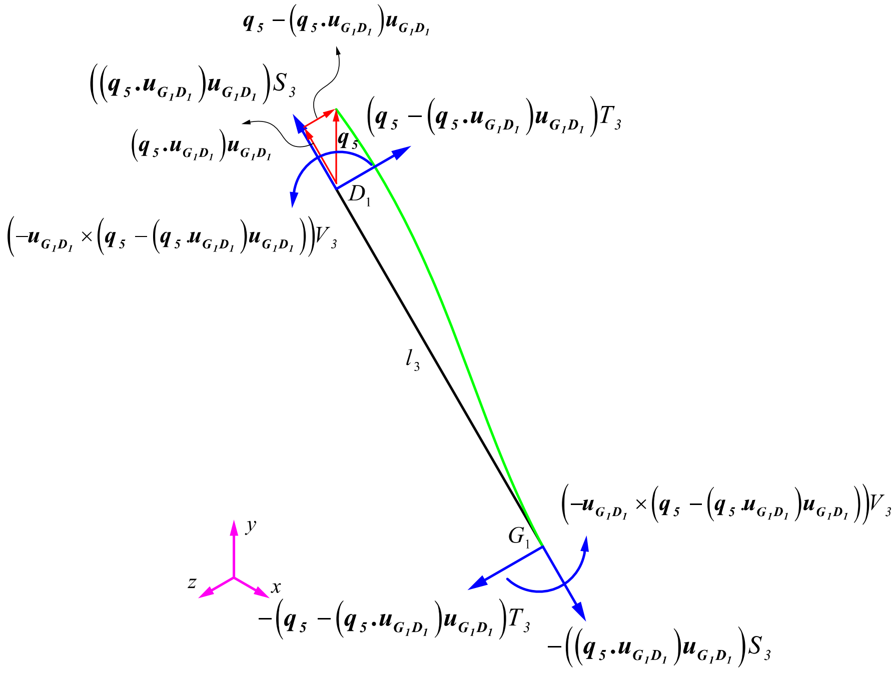

$q_5-(q_5.u_{G_1D_1})u_{G_1D_1}$
$((q_5.u_{G_1D_1})u_{G_1D_1})S_3$
$(q_5-(q_5.u_{G_1D_1})u_{G_1D_1})T_3$
$(q_5.u_{G_1D_1})u_{G_1D_1}$
q_5
D_1
$(-u_{G_1D_1}\times(q_5-(q_5.u_{G_1D_1})u_{G_1D_1}))V_3$
l_3
$(-u_{G_1D_1}\times(q_5-(q_5.u_{G_1D_1})u_{G_1D_1}))V_3$
G_1
y
z
x
$-(q_5-(q_5.u_{G_1D_1})u_{G_1D_1})T_3$
$-((q_5.u_{G_1D_1})u_{G_1D_1})S_3$

(**c**)

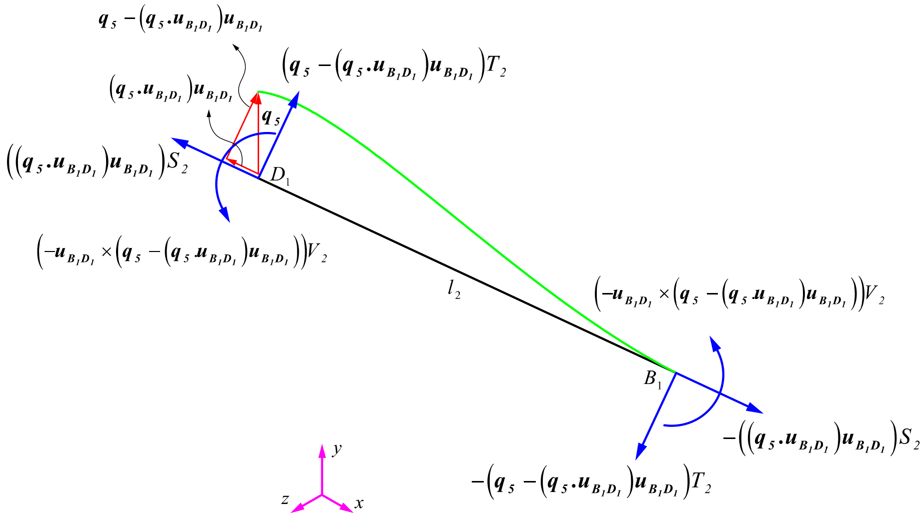

(**d**)

Figure S6. Deformations of (**a**) strut EF, (**b**) strut CD, (**c**) strut DG, and (**d**) strut BD due to $\boldsymbol{q_5} = \mathbf{1}$.

S2. More details on Results

S.2.1. Effect of φ and θ on the Relative Density of Re-Entrant Unit Cell

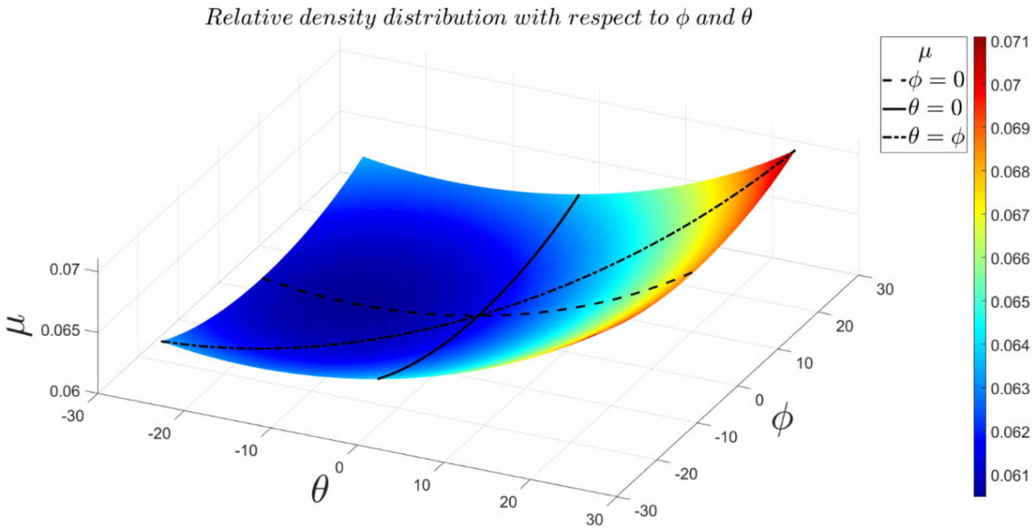

Figure S7. Variation of relative density with respect to θ and φ.

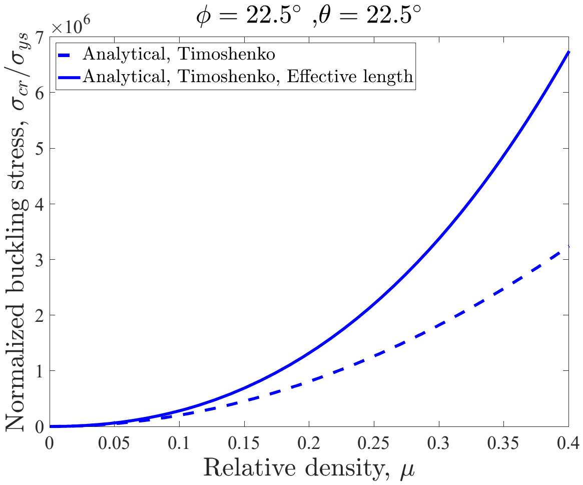

Figure S8. Buckling critical stress of general idealized 3D re-entrant unit cell with and without considering the effective length.

S.2.2. Effect of φ and θ on the Mechanical Properties of Re-Entrant Unit Cell

To this aim, two particular cases with similar strut radii ($r_1 = r_2 = r_3 = 0.14b$) and also $l_1 = b$ have been considered. In the first case, angle θ is varied from 0° to 25°while φ is kept constant at 25°. In the second case, the opposite is done and the results of both the particular cases are shown in Figure S9. The figures show that for all the ranges of θ and φ, both the analytical models which do not consider the over-lapping effect, particularly the model based on Timoshenko beam theory, have very good agreement with the results of the FE models made from beam elements model.

For the case of constant φ = 22.5°, the re-entrant structure has positive Poisson's ratio for $\theta = 0°$ to $\theta = 8.5°$, and then by increasing the value of θ, the Poisson's ratio becomes negative (Figure S9a). Theoretically and by neglecting the overlapping effect, the maximum negative Poisson's ratio value can be obtained as $\nu = -0.45$. However, taking the effective length into account decreases the maximum negative Poisson's ratio for this case to $= -0.127$.

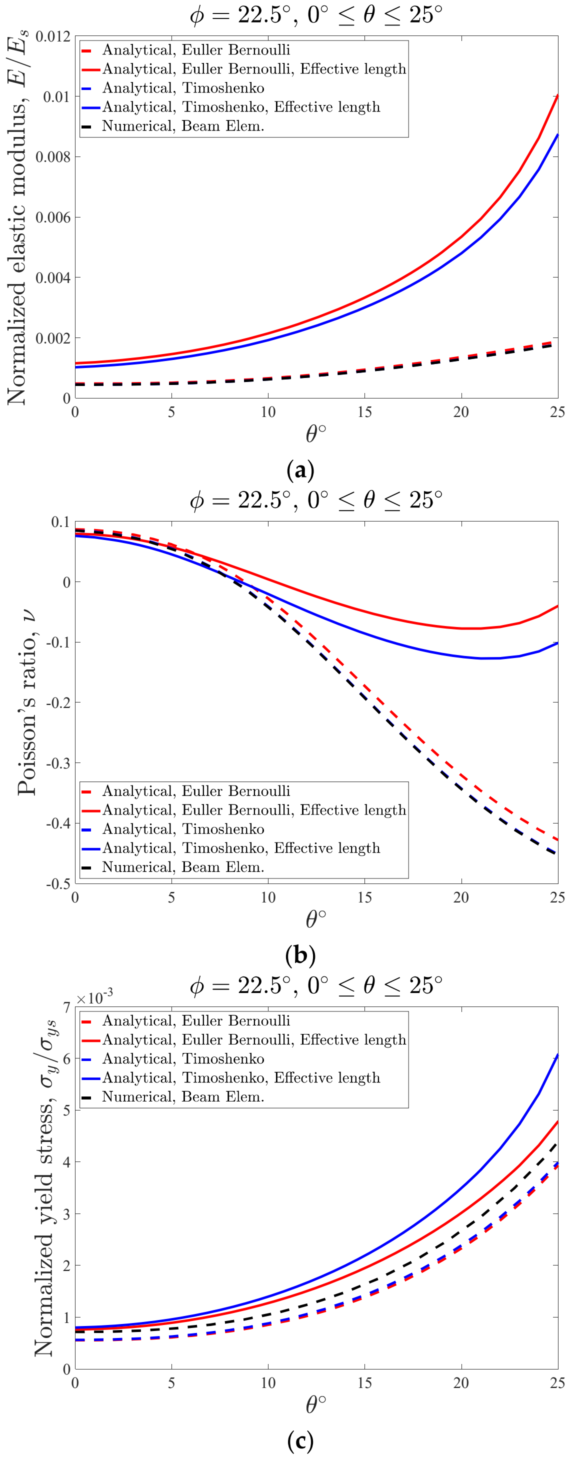

Figure S9. Mechanical properties variations of structure with $\varphi = 22.5°$ and $r/b = 0.14$ with respect to θ: (**a**) elastic modulus, (**b**) Poisson's ratio, and (**c**) yield stress.

For the case of constant $\theta = 22.5°$, throughout the whole range of φ from 0° to 25°, the Poisson's ratio of the analytical model which neglects the overlapping effect remains negative and its value changes from $\nu = -0.23$ to $\nu = -0.42$ (Figure S10a). However, taking the effective length into account decreases the maximum negative Poisson's ratio for this case to $\nu = -0.112$. It is interesting to see that by considering the overlapping effect, Poisson's ratio value remains almost constant for all values of φ (Figure S10a).

By increasing the value of θ at constant $\varphi = 22.5°$, the normalized elastic modulus and normalized yield stress of structure increase. Moreover, considering the effective length effect increases the normalized elastic modulus up to four-

fold at $\theta = 25°$ (Figure S10b). However, an increase in the normalized yield stress values due to consideration of effective lengths is relatively non-significant (Figure S10c). In contrast, at constant $\theta = 22.5°$, increasing the φ angle decreases the normalized elastic modulus and normalized yield stress of structure.

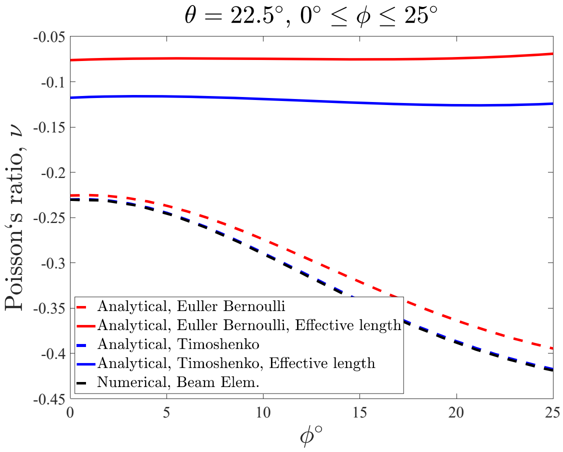

(**a**)

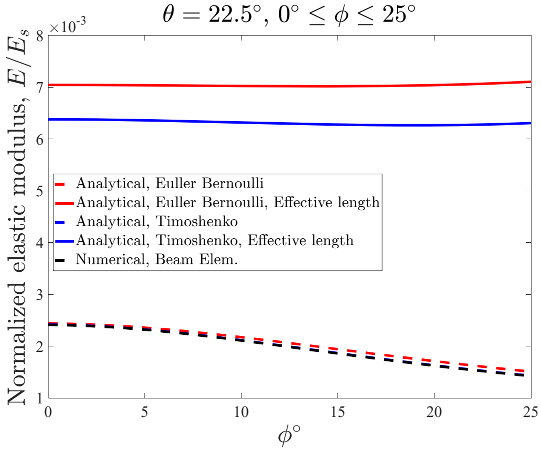

(**b**)

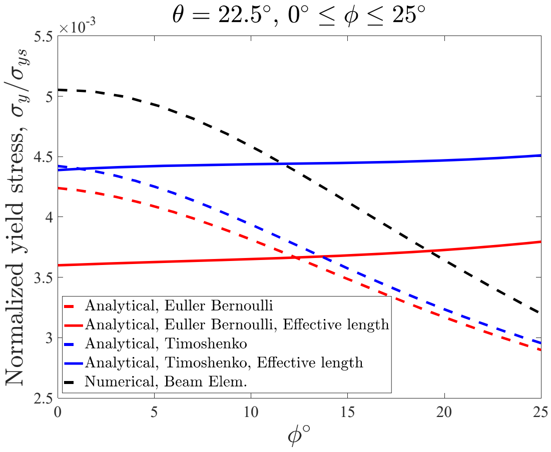

(**c**)

Figure S10. Mechanical properties variations of structure with $\theta = 22.5°$ and $r/b = 0.14$ with respect to φ: (**a**) elastic modulus, (**b**) Poisson's ratio, and (**c**) yield stress.

S.2.3. Comparison between 3D General Re-Entrant Unit Cell and Lightened Re-Entrant Unit Cell

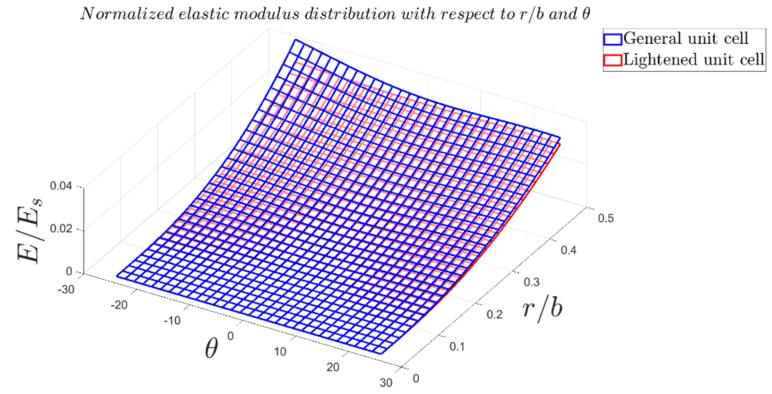

(**a**)

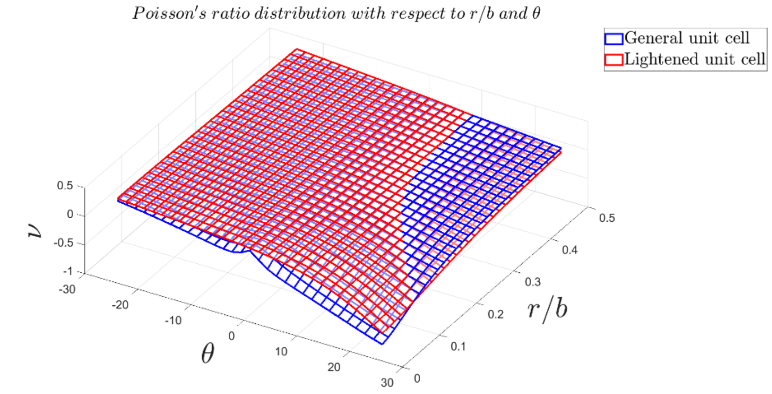

(**b**)

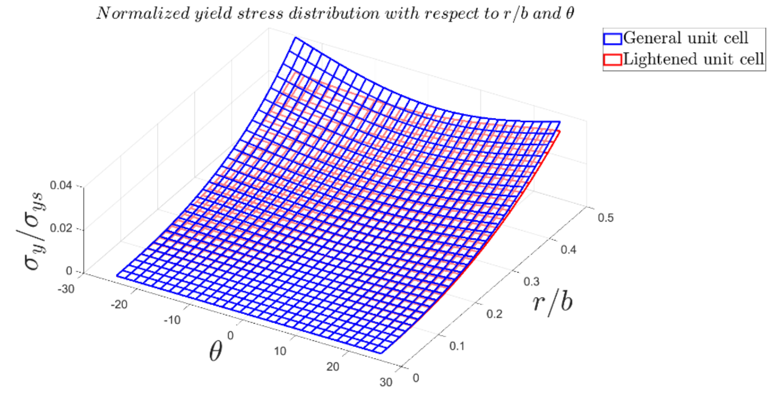

(**c**)

Figure S11. Variation of mechanical properties for general and lightened unit cell with respect to r/b and θ parameters: **a**) normalized elastic modulus, **b**) Poisson's ratio, and **c**) normalized yield stress.

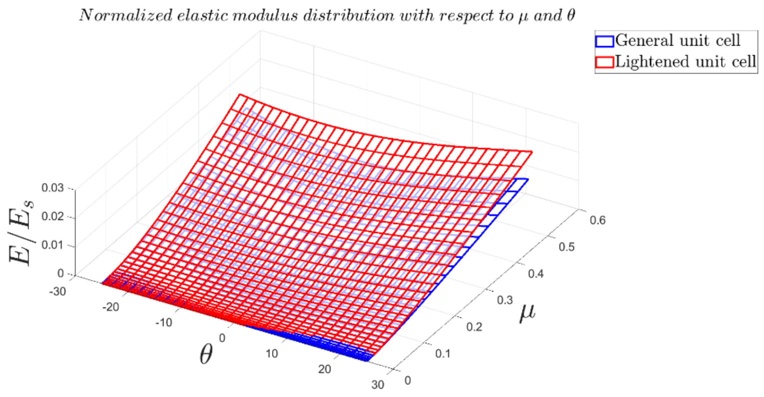

(**a**)

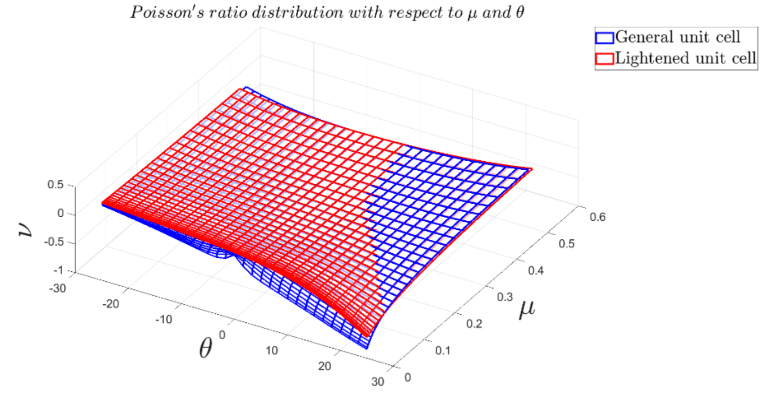

(**b**)

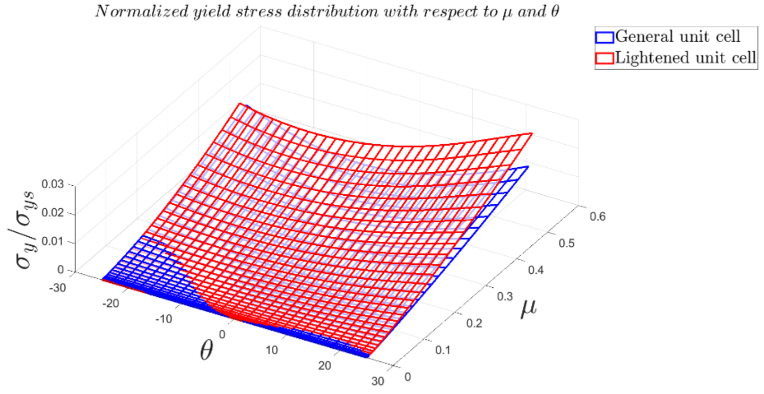

(**c**)

Figure S12. Variation of mechanical properties for general and lightened unit cell with respect to μ and θ parameters: **a**) normalized elastic modulus, **b**) Poisson's ratio, and **c**) normalized yield stress.

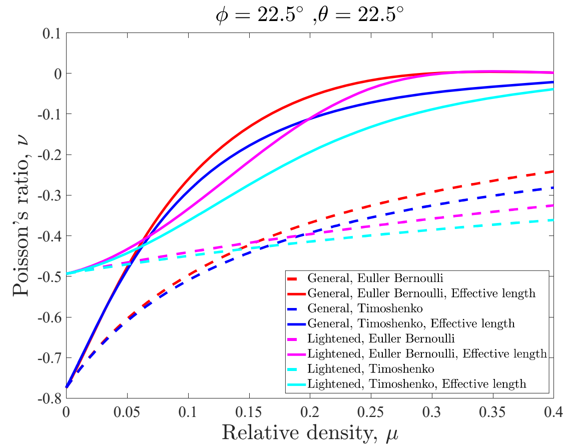

(**a**)

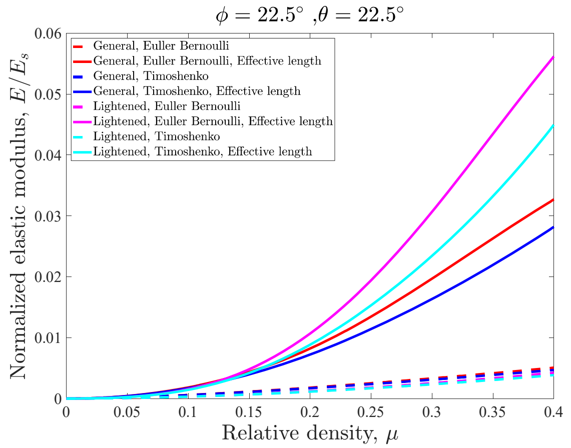

(**b**)

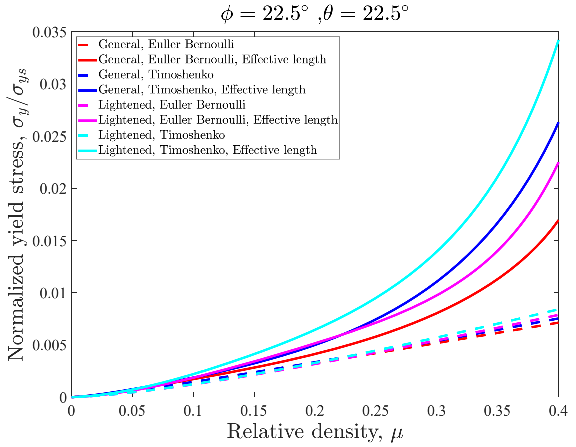

(**c**)

Figure S13. Mechanical properties curves of general and lightened 3D re-entrant structures with $\varphi = 22.5$, $\theta = 22.5$: (**a**) Poisson's ratio, (**b**) elastic modulus, and (**c**) yield stress.

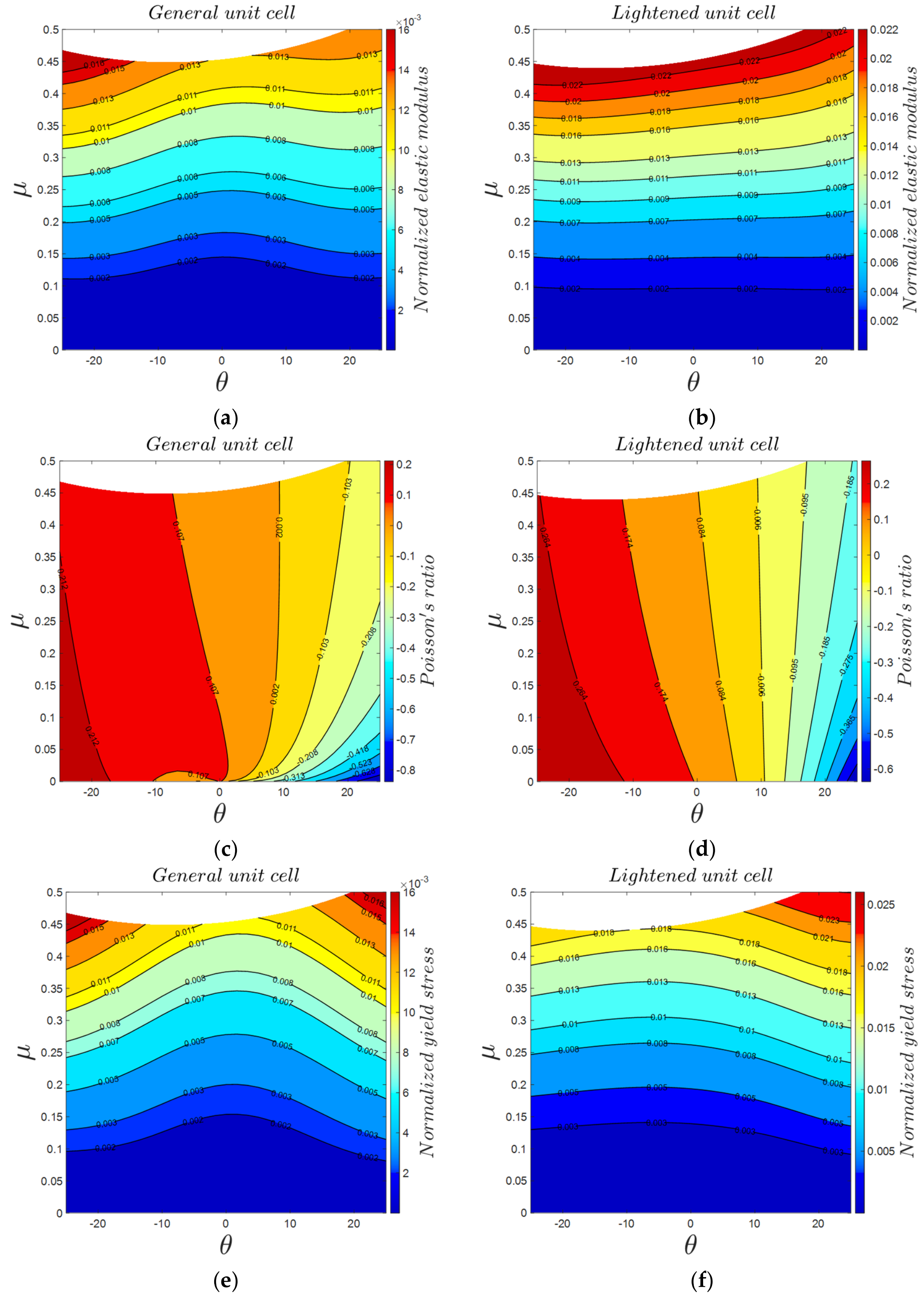

Figure S14. Contour plots of mechanical properties for general (left) and lightened (right) unit cell with respect to μ and θ parameters: **a**,**b**) normalized elastic modulus, **c**,**d**) Poisson's ratio, and **e**,**f**) normalized yield stress.

S.2.4. Numerical Results Based on Beam 189 Elements

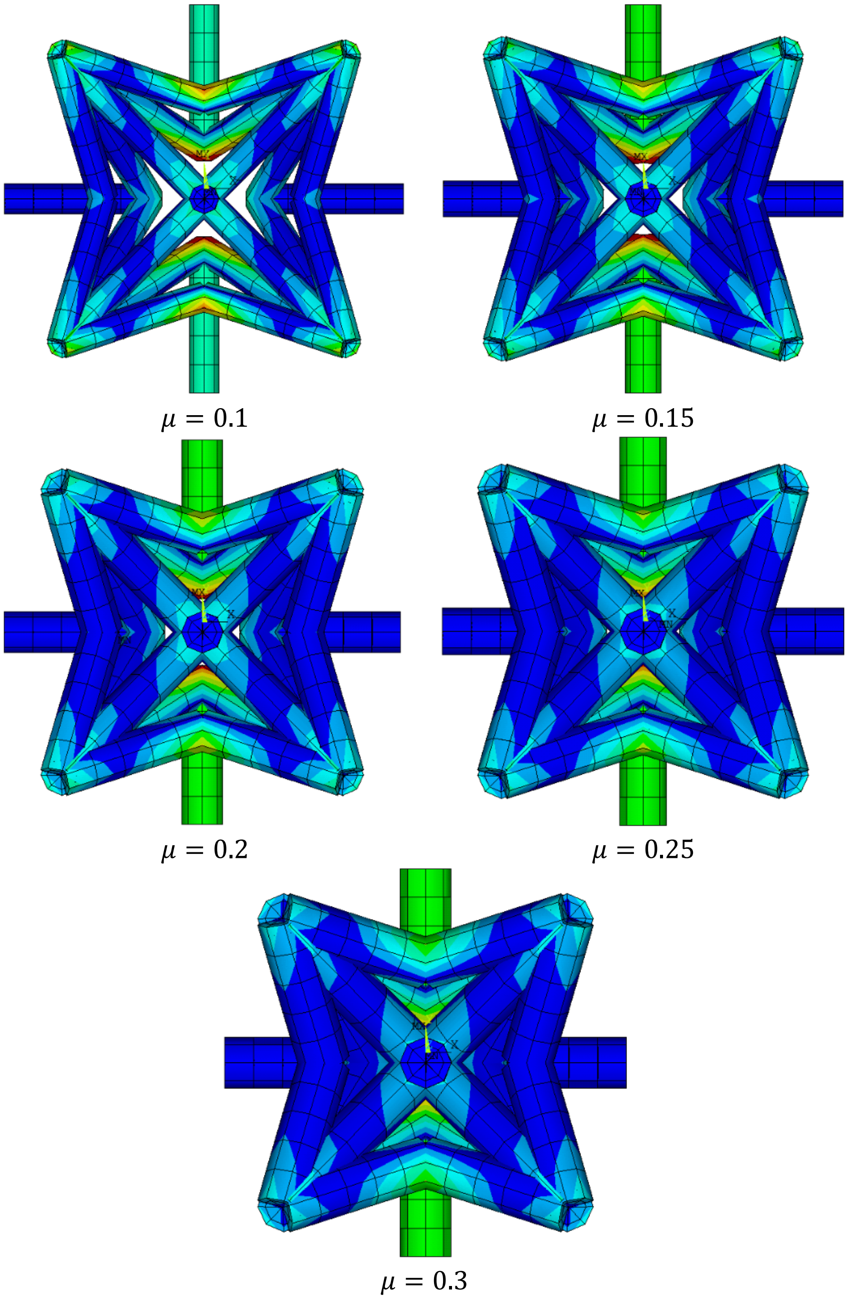

Figure S15. Von Mises equivalent stress (Beam 189 elements).

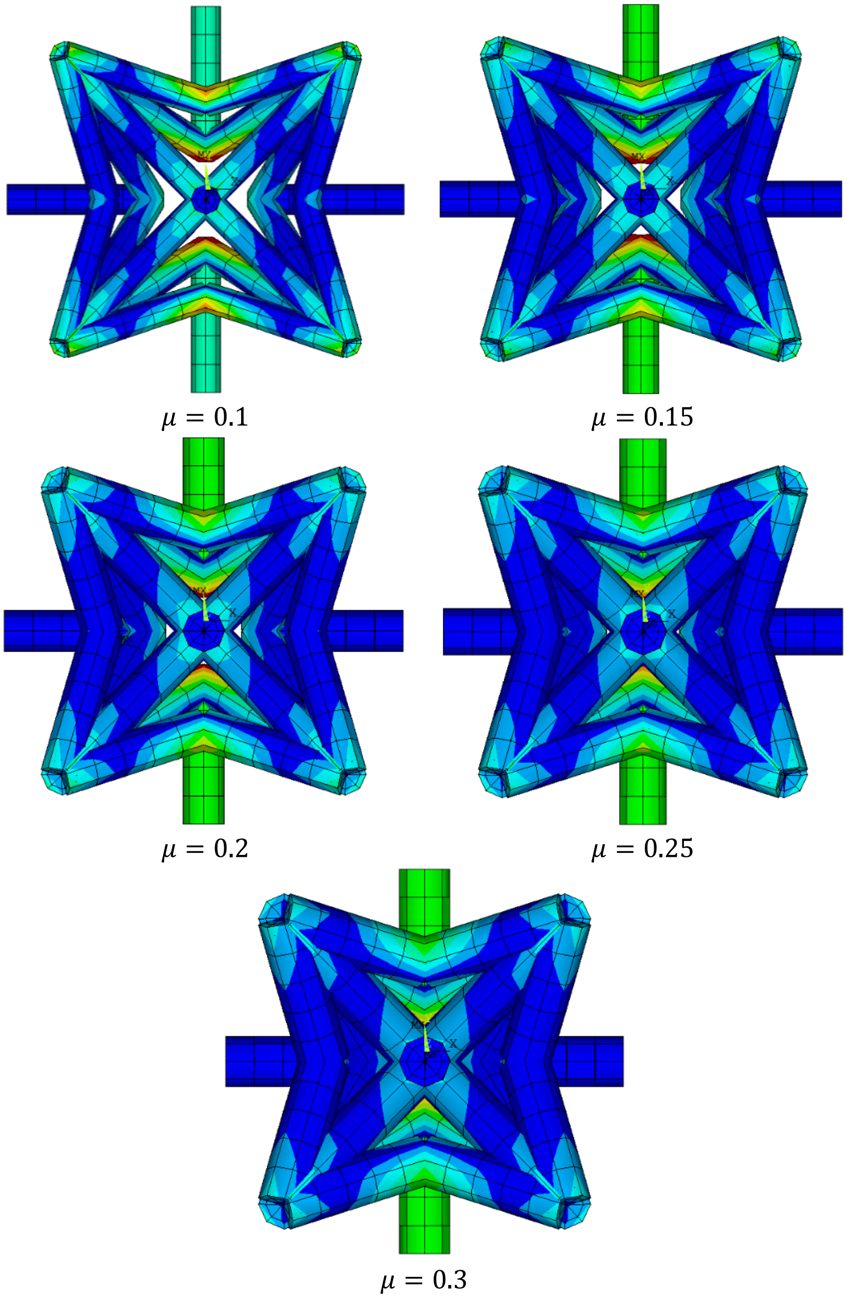

Figure S16. Von Mises equivalent strain (Beam 189 elements).

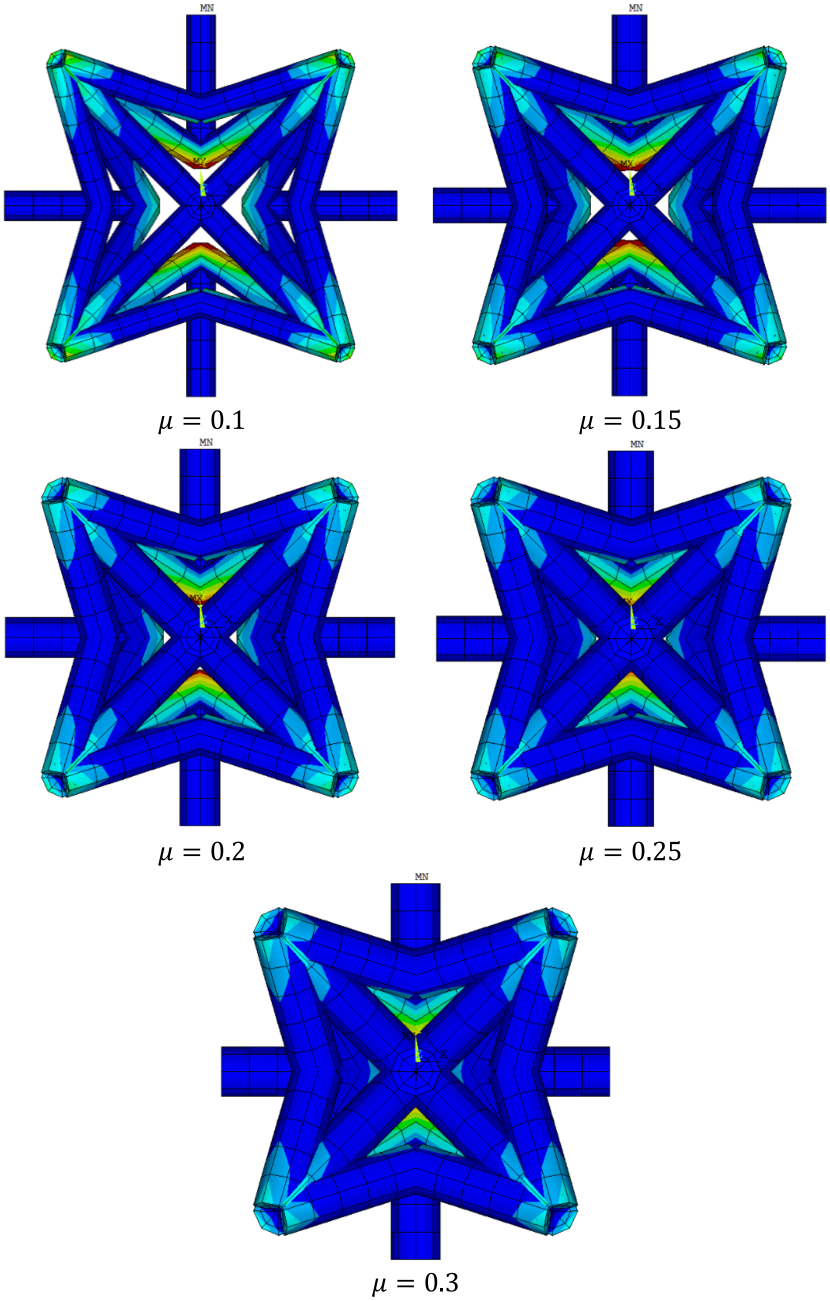

Figure S17. First principal stress (Beam 189 elements).

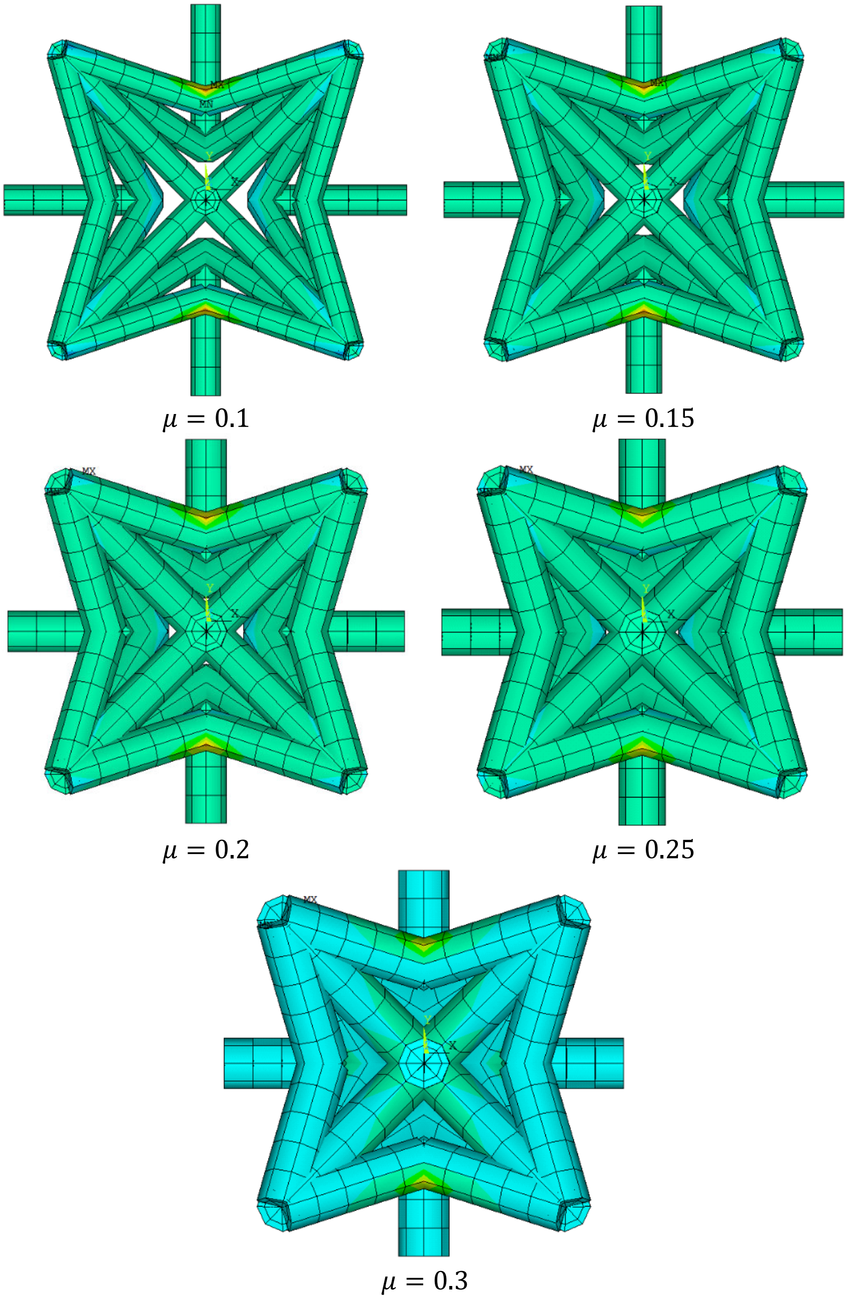

Figure S18. Second principal stress (Beam 189 elements).

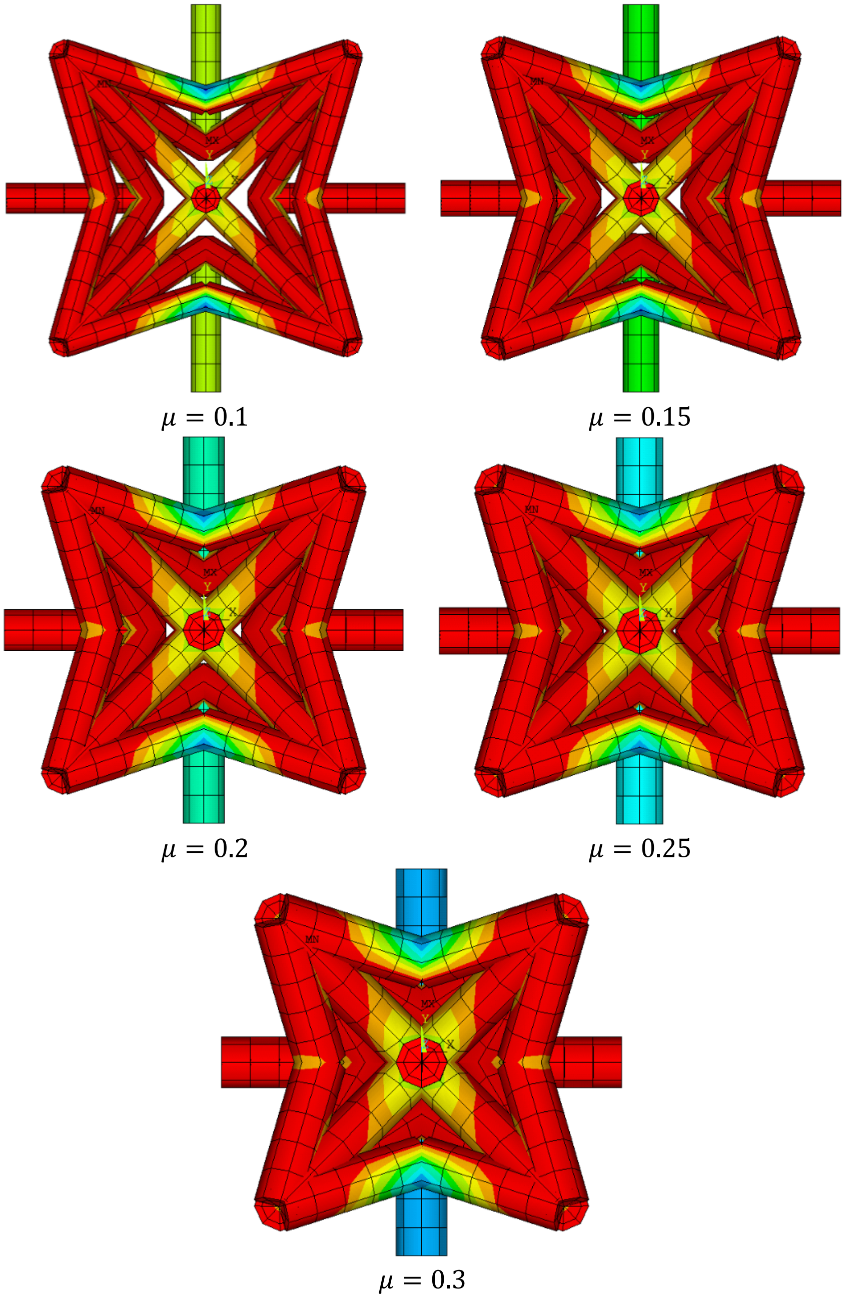

Figure S19. Third principal stress (Beam 189 elements).

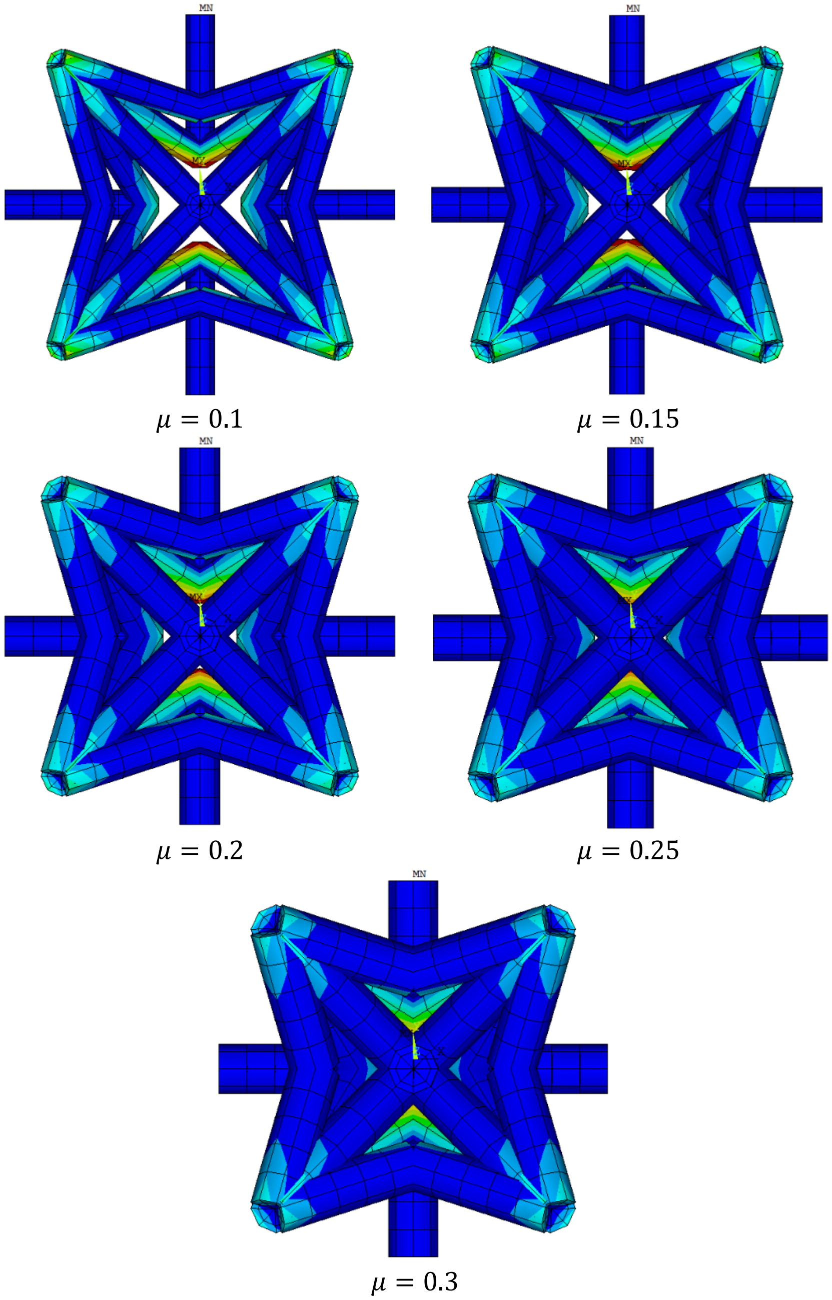

Figure S20. First principal strain (Beam 189 elements).

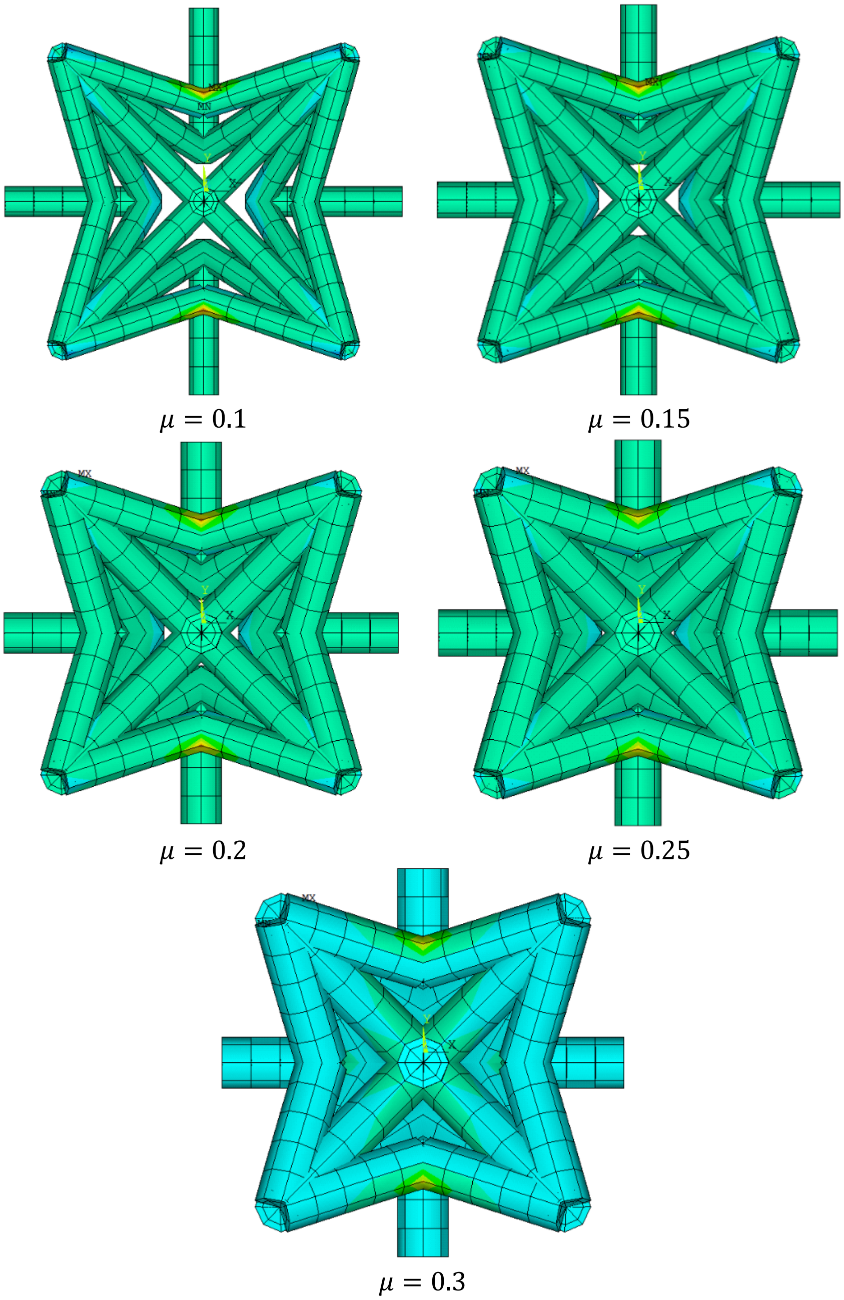

Figure S21. Second principal strain (Beam 189 elements).

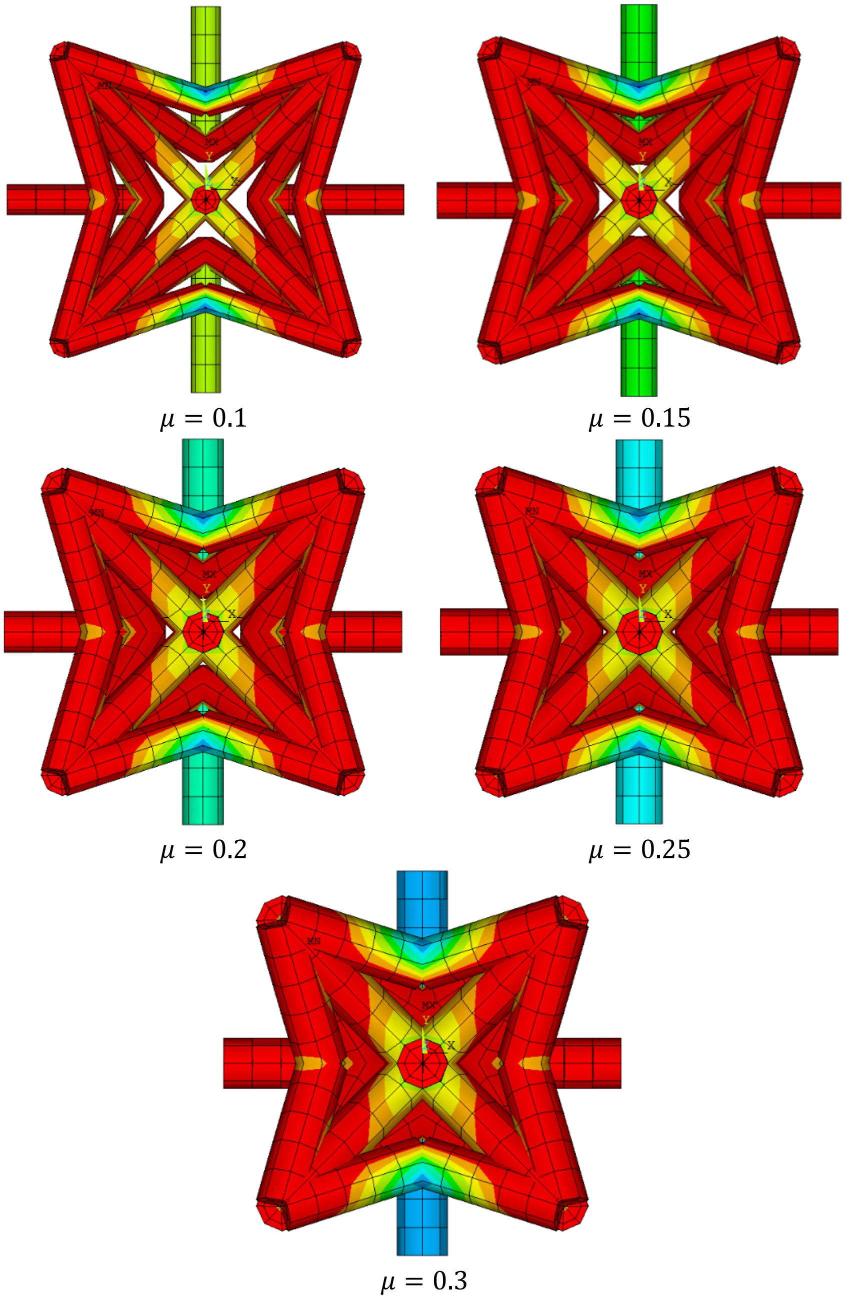

Figure S22. Third principal strain (Beam 189 elements).

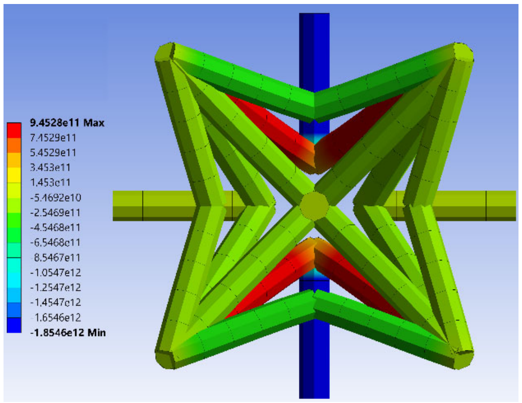

$\mu = 0.1$

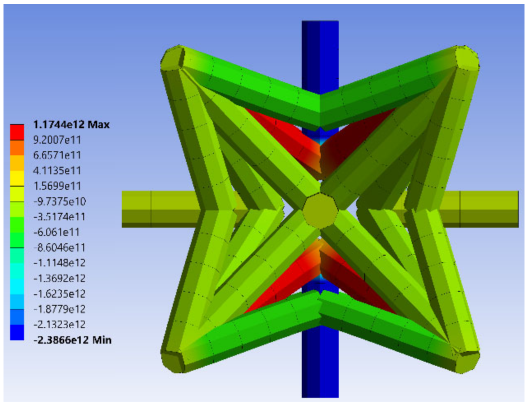

$\mu = 0.15$

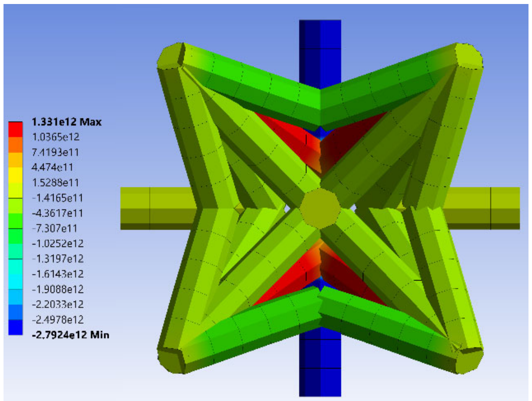

$\mu = 0.2$

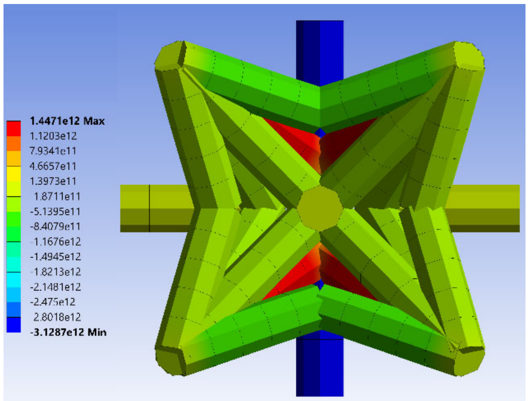

$\mu = 0.25$

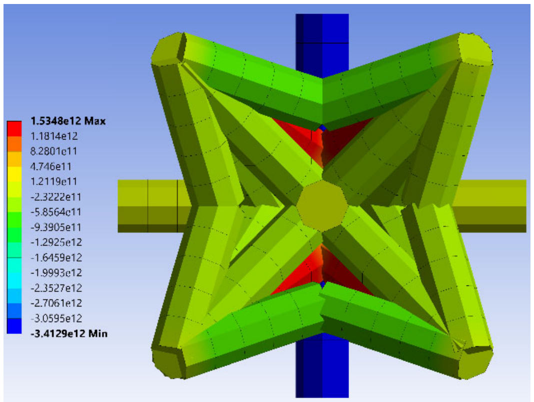

$\mu = 0.3$

Figure S23. Normal stress distribution in the beam elements of models made of BEAM 189 element types.

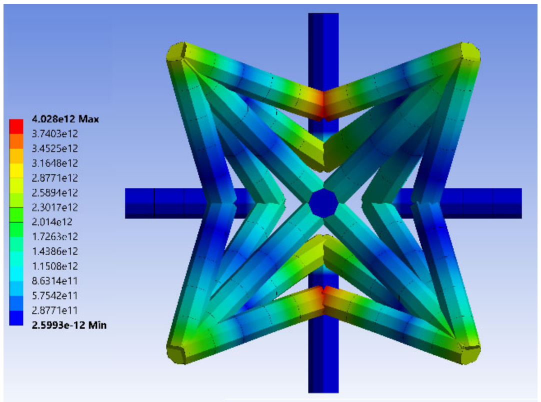

$\mu = 0.1$

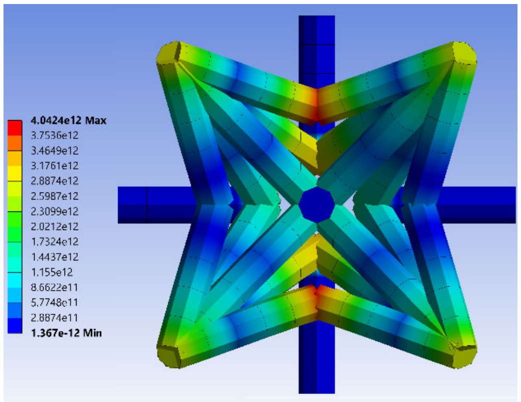

$\mu = 0.15$

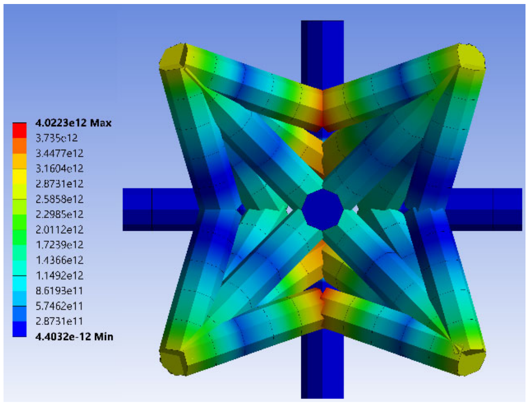

$\mu = 0.2$

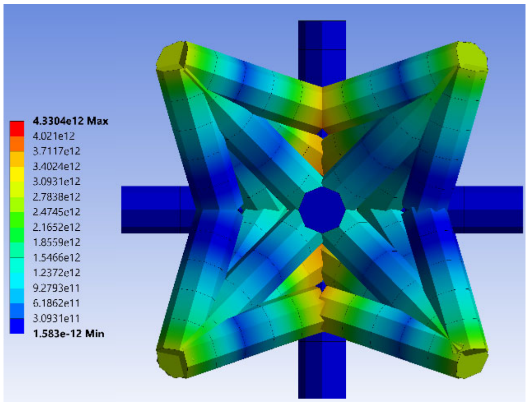

$\mu = 0.25$

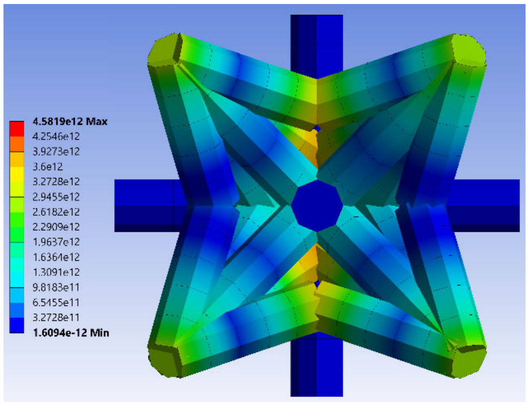

$\mu = 0.3$

Figure S24. Bending stress distribution in the beam elements of models made of BEAM 189 element types.

S.2.5. Numerical Results Based on Solid Elements

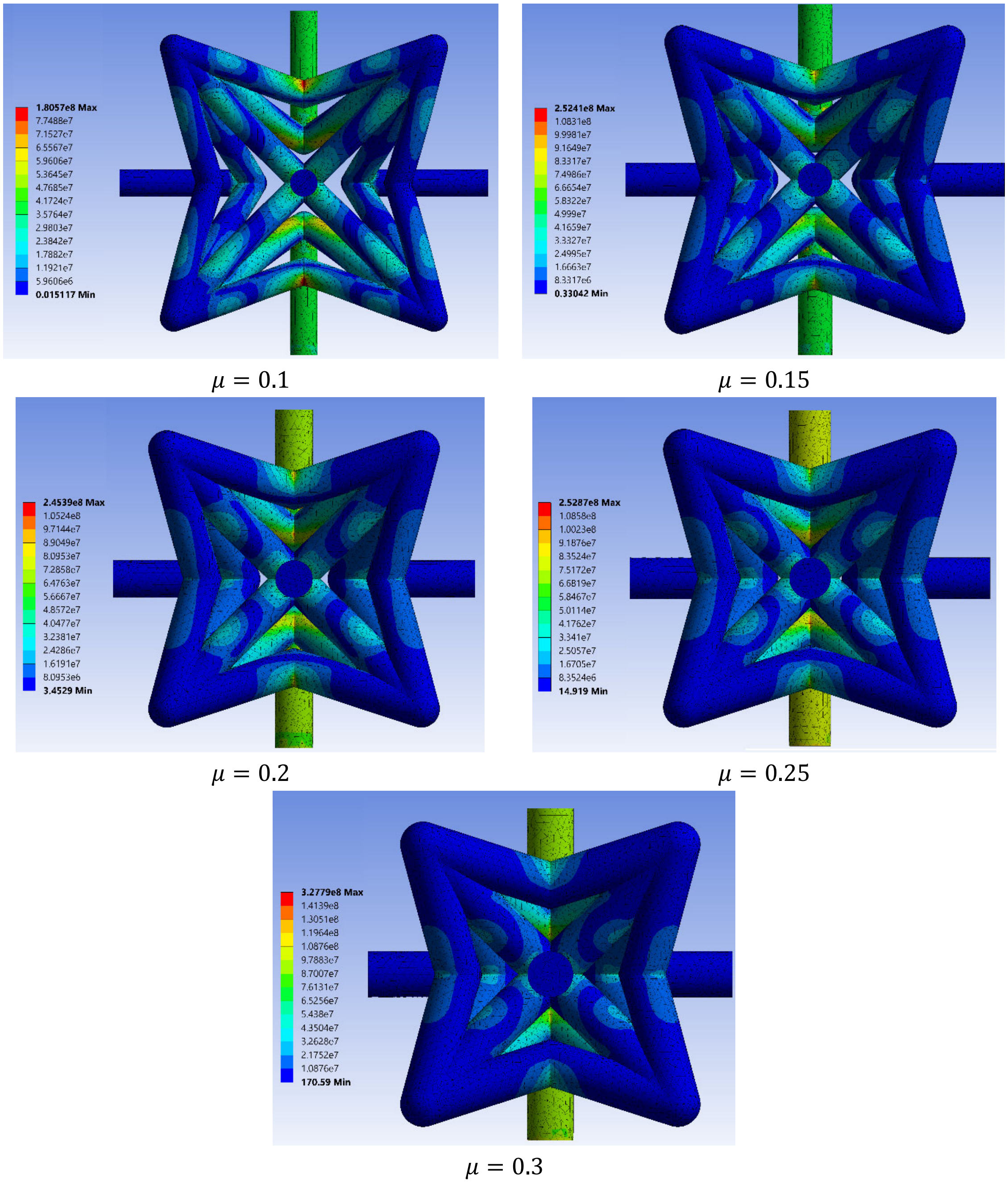

Figure S25. Von Mises equivalent stress for FE models based on solid elements.

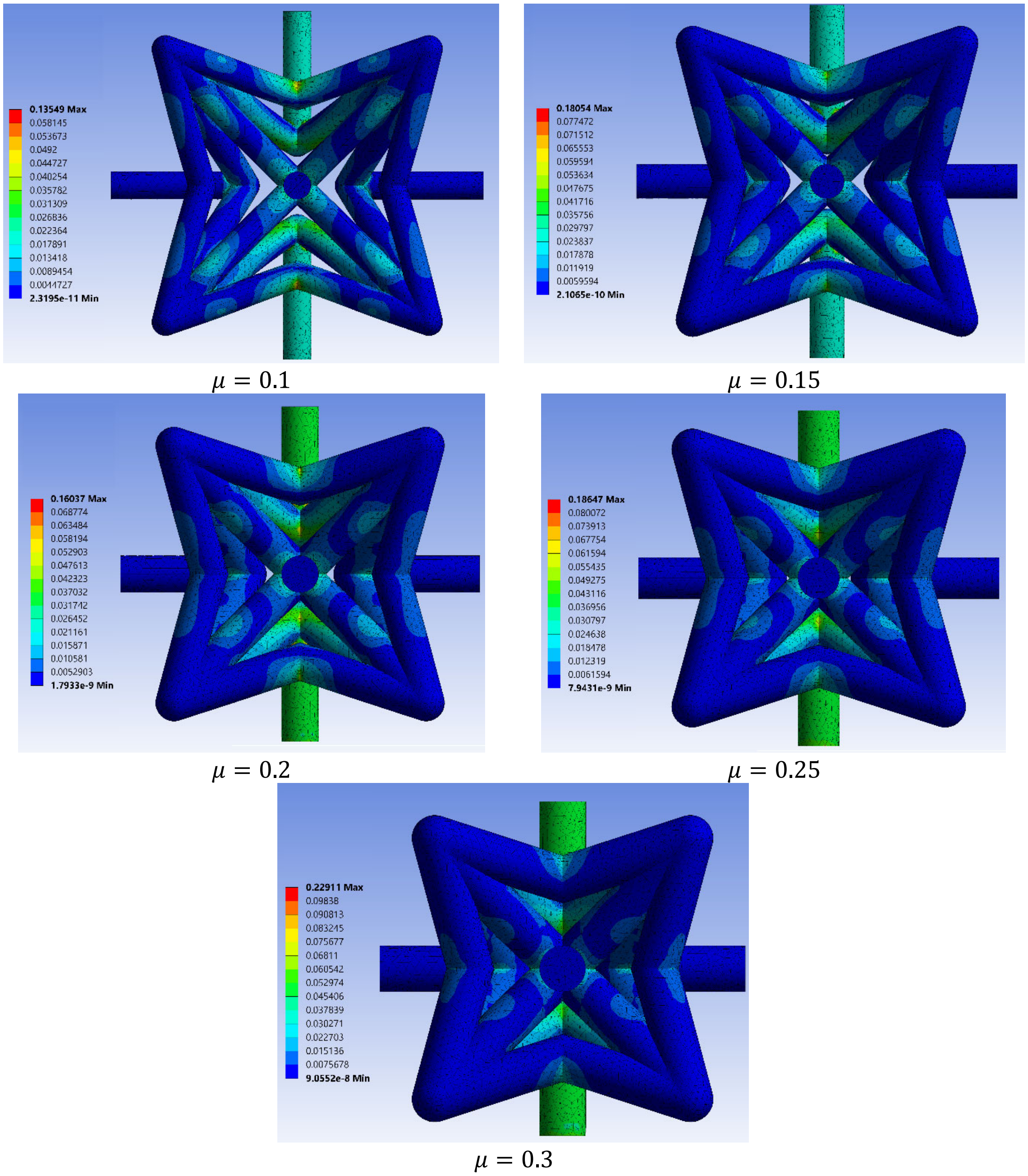

Figure S26. Von Mises equivalent strain for FE models based on solid elements.

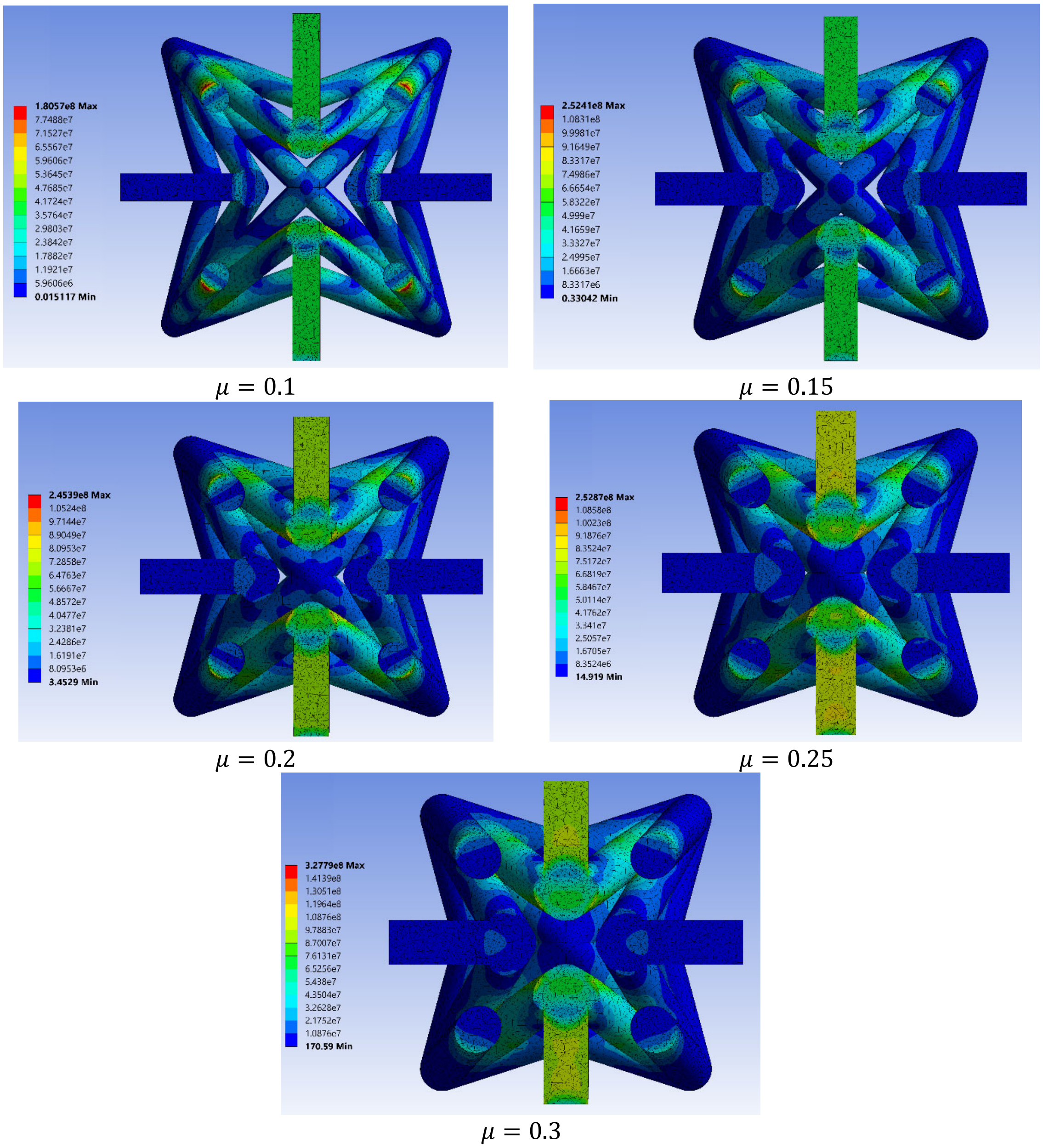

Figure S27. Von Mises equivalent stress for FE models based on solid elements. The unit cells are cut from the central plane for better visualization.
